# Targeting SOD1 via RNAi with PEGylated graphene oxide nanoparticles in platinum-resistant ovarian cancer

**DOI:** 10.1038/s41417-023-00659-2

**Published:** 2023-08-15

**Authors:** Attila Szénási, Enakshi Sivasudhan, Hong Du, Peizhuo Zhang, Jie Huang, Zhijun Zhang, Sonia Rocha, Mu Wang

**Affiliations:** 1 Academy of Pharmacy, Xi’an Jiaotong-Liverpool University, Suzhou, Jiangsu 215123 China; 2 Department of Biological Sciences, Xi’an Jiaotong-Liverpool University, Suzhou, Jiangsu 215123 China; 3 Department of Molecular Physiology and Cell Signalling, Institute of Systems, Molecular and Integrative Biology, University of Liverpool, Liverpool, L69 7ZB UK; 4 Department of Clinical Infection, Microbiology and Immunology, Institute of Infection, Veterinary and Ecological Sciences, University of Liverpool, Liverpool, L69 7ZB UK; 5 Suzhou GenePharma, Suzhou, Jiangsu 215123 China; 6 Key Laboratory of Nano-Bio Interface, Division of Nanobiomedicine, Suzhou Institute of Nano-Tech and Nano-Bionics, Chinese Academy of Sciences, Suzhou, Jiangsu 215123 China

**Keywords:** Cancer therapeutic resistance, Drug discovery

## Abstract

Acquired platinum resistance poses a significant therapeutic impediment to ovarian cancer patient care, accounting for more than 200,000 deaths annually worldwide. We previously identified that overexpression of the antioxidant superoxide dismutase 1 (*SOD1*) in ovarian cancer is associated with a platinum-resistant phenotype via conferring oxidative stress resistance against platinum compounds. We further demonstrated that enzymatic inhibition using small-molecule inhibitors or silencing of *SOD1* via RNA interference (RNAi) increased cisplatin sensitivity and potency in vitro. We launched this study to explore the potential therapeutic applications of *SOD1* silencing in vivo in order to reverse cisplatin resistance using a graphene-based siRNA delivery platform. PEGylated graphene oxide (GO) polyethyleneimine (GO^PEI^-mPEG) nanoparticle was complexed with *SOD1* siRNA. GO^PEI^-mPEG-siSOD1 exhibited high biocompatibility, siRNA loading capacity, and serum stability, and showed potent downregulation of *SOD1* mRNA and protein levels. We further observed that cisplatin and PEI elicited mitochondrial dysfunction and transcriptionally activated the mitochondrial unfolded protein response (UPR^mt^) used as a reporter for their respective cytotoxicities. *SOD1* silencing was found to augment cisplatin-induced cytotoxicity resulting in considerable tumour growth inhibition in cisplatin-sensitive A2780 and cisplatin-resistant A2780^DDP^ subcutaneous mouse xenografts. Our study highlights the potential therapeutic applicability of RNAi-mediated targeting of *SOD1* as a chemosensitizer for platinum-resistant ovarian cancers.

## Introduction

Ovarian cancer, with an annual worldwide incidence of 313,000 cases and mortality of 207,000, is considered the second most lethal gynaecological malignancy [1]. Despite the favourable overall survival when detected in early stage 1—attributed principally to its asymptomatic disease progression—most cases are diagnosed in advanced stages 2 and 3 with a 5-year survival of 31%. The overall global survival has only increased modestly in previous decades due to the constrained availability of treatment options and the clinically acquired chemotherapy resistance [2]. Despite most patients responding to debulking surgery and combinational treatment with platinum and taxols, almost half the patients eventually develop recurrence and become resistant or refractory to additional platinum-based therapeutic interventions [2].

Despite their adverse side effect profile, platinum compounds are still considered potent as the first-line drug of choice as mono- or synergistic therapy for many solid tumours [3–6]. Besides nuclear DNA adduct formation and subsequent induction of apoptotic signalling, platinum drugs also elicit mitochondrial dysfunction through oxidative stress and mitochondrial DNA (mtDNA) damage [7, 8]. Contingent on the intrinsic platinum sensitivity of the respective malignancy, platinum drugs initially exhibit robust therapeutic efficiency. However, the development of acquired platinum resistance of recurrent tumours renders their subsequent clinical applicability ineffective. Lung, prostate, and colorectal cancers are intrinsically resistant to platinum; acquired resistance is also more common in epithelial ovarian cancer [9]. Implicitly, preventing or reversing this well-documented clinical phenomenon could have profound and widespread clinical therapeutic benefits.

Intrinsic and acquired platinum resistance have been linked to reduced drug uptake, increased efflux, enhanced detoxification, elevated scavenger levels, increased oxidative stress tolerance, upregulated DNA repair mechanisms, and the reprogramming of cellular metabolism to evade cisplatin-induced death [10, 11]. Cancer cells may exhibit one, or more of the aforementioned peculiar mechanisms that ultimately determine their net sensitivity to platinum [12, 13]. However, to date, the targeting of drug-transporting pathways of *MDR-1*, *ATP-7A/7B*, *CTR1*, *MRP*, and DNA repair pathways, including *BRCA1/2*, *ERCC1*, and *MMR*, provided only modest improvement in survival clinically [14]. In addition, the utilization of *MDR1* inhibitors, including but not limited to Zosuquidar, MK-571, and PSC-833, showed moderate efficacy in terms of platinum chemosensitization in ovarian cancer [15]. Thus far, the exact mechanisms and determinant factors driving platinum resistance have not been fully elucidated. Due to genetic and patient sample heterogeneity, patient-specific expression levels of potentially robust platinum resistance biomarkers make the discovery process burdensome.

In the quest for a novel therapeutic target for cisplatin resistance, we previously identified the ROS-neutralizing *SOD1* to be overexpressed in cisplatin-resistant ovarian cancer cell lines using quantitative label-free comparative proteomics analysis [16]. The 32 kDa Cu^2+^/Zn^2^^+^ *SOD1* is an abundantly expressed intracellular homodimeric metalloenzyme that converts superoxide anions to hydrogen peroxide that is subsequently transformed by catalase to oxygen and water [17]. Superoxide neutralization by *SOD1* is a crucial mechanism in counteracting oxidative damage, as *SOD1* knockout Drosophila exhibits reduced lifespan, infertility, and hypersensitivity to oxidative stress [18]. Other studies concluded that RNAi-mediated *SOD1* silencing provokes senescence in human fibroblasts and induces apoptosis in HeLa cells via ROS-mediated induction of *TP53* [19]. Further, *SOD1* plays an essential role in cytoplasmic *NRF2*-mediated adaptation to oxidative stress and in the mitochondria via UPR^mt^ [20]. Thus, our previous results suggest the role of *SOD1* overexpression as a defense mechanism of ovarian cancer cells to counteract platinum-induced oxidative stress and modulation of ROS-mediated redox signalling [16]. We further concluded that enzymatic inhibition of *SOD1* using copper/zinc chelating agents of TETA and ATN-224 reversed platinum resistance [21]. Subsequently, to overcome the off-target side effects caused by non-specific small molecule metal chelators, we further corroborated the chemosensitizing effect of *SOD1* downregulation via RNAi in vitro [22].

RNAi is a validated gene therapeutic method to control post-transcriptional gene regulation in various disease states [23]. However, the exogenous introduction of therapeutic siRNA for in vivo applications faces numerous obstacles, including easy degradation, short half-life, filtration by the kidneys, poor cellular uptake due to inherent negative charge, structural instability, and degradation by RNases [24]. Therefore, a meticulous design of any siRNA delivery system is fundamental to fully capture the potential of siRNA therapeutics. Non-viral gene delivery vectors such as cationic lipids, cationic polymers, and polysaccharides, in particular, have gained momentum due to their efficacy and biosafety compared to viral vectors for delivering DNA or RNA cargo into the cells [25].

The two-dimensional graphene oxide (GO), due to its convenient applicability for non-covalent or covalent functionalization via abundant epoxy, carboxyl, and hydroxyl surface groups, has been widely used in combination with various cationic polymers for siRNA and gene delivery [26, 27]. GO is also highly biocompatible in most in vitro and in vivo test systems due to its high colloidal stability, water dispersibility, and surface-to-volume area ratio [28]. We previously synthesized graphene-based siRNA, small molecule, and combined drug delivery systems for various applications [29]. Consequently, in this study, a novel graphene-based nanoparticle platform (GO^PEI^-mPEG) was prepared by sequential coupling of nano-graphene oxide with cationic polymer polyethyleneimine (PEI) to achieve *SOD1* siRNA delivery and further with polyethylene glycol (PEG) to increase biocompatibility and control surface charge. In addition, the incorporation of GO, due to the fixed lateral dimensions of the 2D sheets, can allow for improved control of a more uniform and reproducible final hydrodynamic nanoparticle size, compared to using PEI-PEG polyplexes alone [30, 31]. Our study aimed to evaluate the in vivo chemosensitizing efficacy of *SOD1* siRNA by GO^PEI^-mPEG delivery system in cisplatin-resistant mouse xenograft models.

## Materials and methods

### Nanoparticle preparation

#### Graphene oxide (GO) synthesis

GO was prepared with a modified Hummer’s method [32]. The mixture of NaCl (35 g) and native graphite flakes (1 g, Alfa Aesar, Haverhill, MA, USA) was ground with a mortar until the colour of the mixture turned grey, then dissolved in deionized water (DIW). NaCl was extracted by multiple washing steps and ultracentrifugation at 8000 rpm for 5 min. The product was dried at 90 °C for 12 h and stirred in a three-necked bottle with H_2_SO_4_ (23 mL) for 8 h. In an ice bath, KMnO_4_ (3 g) was added to the mixture keeping the temperature below 20 °C, until the colour turned dark green. The solution was further stirred in a dimethyl-silicone oil bath at 38 °C for 30 min and at 70 °C for an additional 45 min until the colour of the solution turned dark brown. The mixture was diluted with DIW (5 mL) and stirred for 10 min. Next, the solution was diluted with DIW (40 mL) and stirred for 15 min at 100 °C. Subsequently, the mixture of DIW (10 mL) and H_2_O_2_ (10 mL) was added and stirred for 10 min, and the final product was washed twice with 5% HCl (50 mL) and numerous times with DIW via ultracentrifugation. The prepared GO batch was dialyzed using 100 kDa dialysis bags. Next, GO (8 mL) was washed twice with DIW, and the –COOH groups were activated by adding NaOH (1.8 g). Finally, the suspension was diluted with DIW up to 15 mL and stirred at 55 °C for 4 h. Next, the solution was neutralized by adding HCl (5 mL), and the mixture was washed multiple times until the pH became neutral. Following a 30-min bath sonication and centrifugation at 13,000 rpm for 5 min, the concentration of the supernatant was measured with an Evolution 201 UV–Vis spectrophotometer (Thermo-Fisher Scientific, Carlsbad, CA, USA).

### GO^PEI^ synthesis

The conjugation of COOH groups of GO and primary amines of PEI was achieved with zero-length carboxyl to amine carbodiimide (EDC) cross-linking. Carboxyl groups were activated with EDC forming an active but unstable O-acylisourea intermediate displaced by nucleophilic attack from the primary amines of PEI. These primary amines formed an amide bond with the activated carboxylic groups. In brief, GO (5 mg) was diluted up to 15 mL in phosphate-buffered saline (PBS) and was bath sonicated for 30 min in ice before adding 30, 60, or 90 mg of PEI^25 kDa^ (Sigma-Aldrich, St. Louis, MO, USA) dissolved in 200 µL of PBS (HyClone Laboratories Inc, Logan, UT, USA). Following 5 min of sonication, NaOH (20 µL) and 5 mg of Pierce™ Premium Grade EDC [1-Ethyl-3-(3-dimethyl aminopropyl) carbodiimide] (Thermo-Fisher Scientific) were added, respectively, then sonicated for 5 min and stirred for 30 min. GO^PEI^ was prepared using three different PEI concentrations; 30, 60, and 90 mg/mL corresponding with GO^PEI1X^, GO^PEI2X^, GO^PEI3X^, respectively. Next, 10 mg of EDC in 400 µL PBS was added under continuous stirring, sonicated for 30 min, and then stirred overnight. The final EDC concentration was 1 mg/mL in a 10-fold molar or weight excess to GO. The prepared GO^PEI^ nanoparticles were purified with ultrafiltration using a 100 kDa Amicon Ultra-15 centrifugal filter (Merck Millipore Ltd., Darmstadt, Germany).

### GO^PEI^-mPEG synthesis

Methyl-PEG (2000 kDa, 5 mg) (Xi'an Ruixi Biological Technology Co., Ltd, Shaanxi, China) in PBS was diluted to 15 mL and sonicated for 5 min. NaOH (20 µL) and EDC (5 mg) were added and sonicated for 30 min. Next, GO^PEI^ (30 mg) was added, and the solution was sonicated for 5 min before adding EDC (10 mg), then stirred overnight. The nanoparticle was purified with ultrafiltration using a 100 kDa filter (Millipore Sigma, Burlington, MA, USA).

### Nanoparticle characterisation

#### Atomic force microscopy (AFM)

The size and surface morphology of GO and GO^PEI^ were characterized with AFM. The nanoparticle samples (200 μL) were transferred onto a 1.5 cm × 1.5 cm muscovite mica sheet and imaged with Veeco Dimension 3100 atomic force microscope (Veeco Instruments, Bruker, MA, USA). The images were analyzed using V700 (Veeco) and Nanoscope v.7.00b19 (Veeco) software.

#### Transmission electron microscopy (TEM)

The lateral size and qualitative structural properties of GO and GO^PEI^ were assessed with TEM. The nanoparticle samples were transferred onto a 20 cm × 20 cm muscovite mica sheet, then imaged by a Tecnai G2 F20 S-Twin transmission electron microscope (Thermo-Fisher Scientific), and analyzed by Digital Micrograph 3.0 (Thermo-Fisher Scientific) software.

#### Dynamic light scattering (DLS)

The average size distribution (ASD), the polydispersity index (PDI), and surface charge (ζ-potential) of GO, GO^PEI^, GO^PEI^-mPEG nanoparticles were determined with DLS. The samples (100 μL) were first filtered, then diluted up to 2 mL with DIW and transferred into a ZEN0112-low volume disposable sizing cuvette for ASD and PDI and into a DTS1060C-Clear disposable zeta cell for ζ-potential analysis. The measurements were performed with a Zetasizer Nanoseries Nano-25 (Malvern, United Kingdom) in batch mode at 25 °C in triplicates with 120 sec equilibration time. The results were analyzed with the Zetasizer (Malvern, United Kingdom) software.

#### Fourier transform infrared (FT-IR) spectroscopy

The confirmation of successful covalent amide bond formation between GO and PEI was determined with FT-IR spectroscopic analysis for GO, GO^PEI^, GO^PEI^-mPEG samples with a Cary 600 series (Agilent Technologies, Santa Clara, CA, USA) FTIR Spectrometer, and all obtained results were analyzed with the Resolution Pro (Agilent Technologies, Santa Clara, CA, USA) and displayed with the GraphPad Prism 7 software (GraphPad Software Inc, Boston, MA, USA).

#### Ultraviolet-visible (UV–Vis) spectroscopy

The nanoparticle absorbance spectra and the siRNA oligonucleotide loading capacity were measured and confirmed with UV–Vis spectroscopy. The GO (0.01 mg/mL), PEI (0.005 and 0.01 mg/mL), GO^PEI^, GO^PEI^-siSCR (scrambled negative control), GO^PEI^-mPEG, and GO^PEI^-mPEG-siSCR samples complexed at w/w ratio of 3:1 (nanoparticle : siRNA) at RT for 30 min and scrambled negative control siRNA (siSCR) only samples (50 μL) were dissolved in DIW (2 mL) in a 10 mm quartz cuvette (3.5 mL). The absorbance spectrum was recorded compared to DIW as a calibration control. The UV–Vis spectra were measured with an Evolution 201 UV–Vis spectrophotometer (Thermo-Fisher Scientific) between 190 and 800 nm in absorbance mode and were analyzed with the Thermo Cue (Thermo-Fisher Scientific) software.

#### Thermogravimetric analysis (TGA)

The composition and PEI surface coating of lyophilized GO and GO^PEI^ nanoparticles were quantitatively assessed with an STA 449 F3 Jupiter® thermal analyzer (Netzsch, Germany). Under inert nitrogen purge (50 mL/min), the samples were heated (25–800 °C, 50 °C/min), and the change of the initial nanoparticle mass (5 mg) was measured as a function of time and temperature. Ceramic crucibles with loosely fitted lids containing the samples were held at 25 °C for 5 min prior to measurements to allow for equilibration between the crucible and the furnace. To correct the bias caused by the buoyancy effect, a blank measurement with an empty ceramic crucible under the same temperature program as the samples were performed. Results were analyzed with the Netzsch Proteus Thermal Analysis software and displayed as a thermogravimetric temperature vs mass curve with the following formula:

$$\left( {{\it{W}}\;{\it{Sample}}\;{\Delta}{\it{t}} - {\it{blank}}} \right)/\left( {{\it{W}}^{\left( {{\it{t}} = 0} \right)} - {\it{blank}}} \right) \times {\it{100}}$$

where *W*^*t=0*^ is the initial sample weight, and *W Sample* ∆*t* is the measured sample weight at different time points.

#### Colloidal stability studies

To assess the dispersibility, stability, and aggregation potential of GO, GO^PEI^, and GO^PEI^-mPEG, the nanoparticles were dissolved in various serum-containing and serum-free salt solutions, including DIW, PBS, normal saline (0,9%), RPMI (HyClone Laboratories Inc, Logan, UT, USA), RPMI with 5% v/v FBS (Biological Industries, Cromwell, CT, USA), RPMI with 10% v/v FBS, 50% v/v FBS and 50% v/v PBS, RPMI with 10% FBS in 1 M HCl (pH = 6.4). As mentioned earlier, the solutions without nanoparticles served as negative controls. All nanoparticles were filtered under sterile conditions before mixing with the solutions in sterilized glass vials with a 0.45 µM pore diameter syringe filter (Merck Millipore Ltd., Darmstadt, Germany) and were left at RT for six months. The aggregation potential of nanoparticles was assessed by inspection at 1, 4, 12, and 24 weeks and by DLS measurements at 4 weeks.

#### Gel retardation assay (GRA)

The nanoparticles' siSCR loading and retention capacity, including GO, GO^PEI^, and Lipofectamine 2000^TM^ (Lipo^2000^), were investigated with GRA. GO was mixed with siSCR at w/w ratios of 2:1, 4:1, 6:1, 8:1, 10:1, 12:1, 14:1, 16:1, 18:1, and 20:1 respectively, while GO^PEI^ and Lipo^2000^ at a w/w ratios of 0.25:1, 0.5:1, 0.75:1, 1:1, 1.25:1, 1.5:1, 1.75:1, 2:1, 2.25:1, 2.5:1, respectively, while siSCR with sense 5′-UUCUCCGAACGUGUCACGUTT-3′ and antisense 5′-ACGUGACACGUUCGGAGAATT-3′ sequences (Suzhou GenePharma, Suzhou, China) served as the negative control in DNase, RNase free Ultrapure^TM^ Distilled Water (Invitrogen, Grand Island, NY, USA). The nanoparticles were left at RT for 30 min to complex with siSCR, then mixed with 5X RNA loading buffer (4 μL) in a total volume of 24 μL. The samples were loaded into an agarose gel (1%) (Sigma-Aldrich) prepared with 15 μL of 10,000 X Gel Red (Biotium Inc, Fremont, CA, USA) in 20X TAE Buffer (Thermo-Fisher Scientific, Carlsbad, CA, USA) and ran for 15 min at 120 V. The gel images were recorded with a ChemiDoc^TM^ MP Imaging System (Bio-Rad) or Gel Doc TM XR+ Molecular Imager (Bio-Rad) with the Image Lab^TM^ Touch v.2.3.0.07 and Quantity One v.4.6.8 software, respectively.

#### Serum stability

The siRNA cargo retention properties and the susceptibility of GO^PEI^ complexed siSCR to RNase degradation were measured with a serum stability assay. Naked siSCR (0.66 µg), 50% FBS, 10%, 30%, 50%, 100% FBS dissolved in PBS, 100% FBS, 4 µg GO^PEI^ (with 1X, 2X, and 3X of conjugated PEI concentrations) in pure FBS, DEPC water and pure FBS, 4 µg PEI (5 mg/mL) in DEPC water were investigated. The nanoparticle samples were complexed with siSCR at a 3:1 w/w ratio and kept at RT for 30 min to allow full complexation. Next, all samples were incubated at 37 °C and at predetermined time points of 0, 0.5, 1, 2, 2.5, 3, 6, 8, 12, 16, 24, and 48 h, respectively, a portion of the respective samples mixed with 5X RNA loading buffer (4 µL) (SolarBio, Beijing, China) was loaded into an agarose gel (1%) containing Gel Red (15 µL), and the samples were run for 10 min at 120 V. The gels were imaged with Gel Doc TM XR+ (Bio-Rad) Molecular Imager and analyzed with Quantity One v.4.6.8 (Bio-Rad) software. The stability of the nanoparticles complexed with FAM-labelled siSCR (siSCR^FAM^) with sense 5′-UUCUCCGAACGUGUCACGUTT3′ and antisense 5′-ACGUGACACGUUCGGAGAATT-3′ sequences (Suzhou GenePharma) in mouse serum was further measured in a fluorescent kinetic study. In a 96-well plate (Corning Inc., Corning, NY, USA) in triplicates, 50 µL of PBS (blank), 1:1 PBS with mouse serum, 1:1 PBS with mouse serum plus siSCR as negative control, GO^PEI^ conjugated siSCR, and Lipo^2000^ complexed siSCR as positive control were measured. The fluorescence was recorded for 8 h at an excitation wavelength of 488 nm and emission wavelength of 520 nm using a Varioskan LUX multimode microplate reader and analyzed using Skanit Re v.4.1 (Thermo-Fisher Scientific) software.

### In vitro assays

#### Cell culture

A2780 cisplatin (CP) sensitive and A2780^DDP^ CP resistant human ovarian epithelial adenocarcinoma cell lines were a kind gift from Sun Yat-sen University Cancer Center (Department of Gynecology, Guangdong, China). A2780 and A2780^DDP^ cell lines were grown in full media consisting of Dulbecco’s Modified Eagle Media (DMEM, HyClone Laboratories Inc, Logan, UT, USA) media supplemented with 10% FBS (Gibco, Thermo-Fisher Scientific, USA) and 1% penicillin/streptomycin. The cell lines were grown in a humidified incubator at 37 °C with 5% CO_2_. The cisplatin-resistant phenotype of A2780^DDP^ was maintained by supplementing 20 µg/ml cisplatin (Sigma-Aldrich) every 4 weeks for 24 h. For cell passaging, the media was removed, and cells were washed twice with PBS, then 5 ml of 0.25% (w/v) trypsin-EDTA (Sigma-Aldrich) was added and incubated in 5% CO_2_ at 37 °C for 6–7 min. Next, 9 mL of full media was added. The cell suspension was centrifuged in a polypropylene conical centrifuge tube (15 mL) with a Sorvall™ ST 16 Centrifuge (Thermo-Fisher Scientific) at RT for 3 min at 800 rpm. Following removing the supernatant, cells were re-suspended and counted manually under an Eclipse TS 100 inverted microscope (Nikon, Japan) with a modified Neubauer hemocytometer using Trypan blue (Invitrogen) to quantify cell viability.

#### Live cell FAM uptake

Based on graphene-based nanomaterials' ability to quench specific fluorophores' fluorescence, siSCR^FAM^ was complexed with GO^PEI^ and GO^PEI^-mPEG, and the cellular uptake and siSCR^FAM^ release in A2780^DDP^ cells were measured at excitation and emission wavelengths of 488 nm and 520 nm, respectively. The nanoparticle samples were pre-complexed with siSCR^FAM^ at RT for 30 min in triplicates in a final volume of 50 µL and were aliquoted into a 96-well plate, while DIW, RPMI media, nanoparticles only, and naked siSCR^FAM^ served as negative controls. Under maintained CO_2_ levels (5%) and temperature (37 °C), the fluorescent signal was recorded for 12 h with a Varioskan LUX (Thermo-Fisher Scientific) plate reader, and the results were analyzed with the SkanIt^TM^ (Thermo-Fisher Scientific) software.

#### Laser confocal microscopy

A2780 and A2780^DDP^ cells (5 × 10^4^) were seeded in collagen-coated (*d* = 35 mm) gamma-irradiated glass bottom micro-well confocal dishes (MaTek Corporation, Ashland, MA, USA) and incubated overnight in a 5% CO_2_ incubator at 37 °C. siSCR^FAM^ (2 µg) dissolved in Opti-MEM (500 µl) reduced serum media (Gibco, Carlsbad, CA, USA) served as the negative control, GO^PEI^ (6 µg) and GO^PEI^-mPEG (6 µg) were complexed with siSCR^FAM^ (2 µg) at w/w ratio of 3:1 for 30 min, and 15 µg of Lipo^2000^ (1 mg/mL) (Thermo Fisher) as a positive control was complexed with siSCR^FAM^ (2 µg) for 30 min, Lipo^2000^ and GO^PEI^ without siSCR^FAM^ were used as negative controls, and cells without treatment as blank controls. Next, the media was removed following overnight incubation with the test samples, the dishes were washed with ice-cold PBS thrice, and serum-free RPMI media (500 µL) was added. The confocal dishes were completely covered with aluminium foil and incubated for 5 h in a 5% CO_2_ incubator at 37 °C. Next, the cells were washed with ice-cold PBS twice and fixed with formaldehyde (3.7%) for 10 min. For cell permeabilization, Triton X-100 (0.2%) (Sigma-Aldrich, St. Louis, MO, USA) was added for 5 min, then washed with PBS thrice. The nuclei of the cells were stained with DAPI (1 mg/ml) nuclear stain (Sigma-Aldrich, St. Louis, MO, USA) dissolved 1:5000 in PBS. Following a 20 min incubation at 40 °C in the dark, the cells were washed with PBS thrice. They were imaged in confocal mode with a Zeiss laser confocal scanning microscope at 10X and 40X resolution objectives. The excitation wavelengths for DAPI and siSCR^FAM^ were 405 and 488 nm, while the emission was recorded in the 460–490 nm and 500–530 nm wavelengths, respectively. The obtained images were analyzed with the Zeiss LSM 880 (Zeiss, Oberkochen, Germany) and ZEN Imaging software 2.3 (Zeiss).

#### Fluorescence-activated cell sorting (FACS)

The siRNA transfection efficiency of the nanoparticles was quantified with FACS. A2780^DDP^ cells (3 × 10^5^/well) were seeded in six-well plates and incubated overnight prior to transfection. The plates were transfected with GO^PEI^ (0.4, 0.8 and 1.2 µg) complexed siSCR^FAM^ (30, 60, 90 nM) at a w/w ratio of 3:1, Lipo^2000^ (positive control) complexed siSCR^FAM^ (30, 60, 90 nM), GO (22 µg) complexed siSCR^FAM^ (90 nM), and blank (media only), naked siSCR^FAM^, GO^PEI^ and Lipo^2000^ served as negative controls. To assess the siRNA transfection efficiency of GO^PEI^-siSCR^FAM^ complexes at 0.2:1, 0.8:1, 1:1, 2:1, 5:1, and 10:1 w/w ratios, GO^PEI^ was mixed with siSCR^FAM^ (40 nM) at RT for 30 min. For transfection, the media was first replaced with serum-free DMEM (500 µL), and the test samples suspended in Opti-MEM (500 µL) were added to the wells dropwise. Following transfection, the plates were incubated at 5% CO_2_ at 37 °C for 5 h, then the media was replaced, and the plates were incubated for at least 48 h until they reached 80% confluency. The adherent cells were harvested by trypsinization and centrifugation and were re-suspended in ice-cold PBS (1 mL). The fluorescence of the cells was measured with a FACSCalibur (BD Biosciences, Franklin Lakes, NJ, USA) flow cytometer by analyzing 10,000 events per measurement. Cells were isolated according to their granularity and size predetermined by the Side Scatter (SSC-H), Forward scatter (FSC-H), and green FAM fluorescence band pass filter (FL1-525 nm) parameters with a BD CellQuest Pro (BD Biosciences) software, and the results were analyzed with the FlowJo software (BD Biosciences).

#### Quantitative real-time polymerase chain reaction (qRT-PCR)

The baseline and time-course *SOD1* mRNA expression levels at 4, 8, 16, 24, 36, and 48 h and the knockdown efficiency of GO^PEI^ and GO^PEI^-mPEG complexed siSOD1 (1–300 nM) at different w/w ratios (0.2:1 to 10:1) along with the effect of GO^PEI^ treatment (1–8 µg/mL) alone on the *SOD1* mRNA expression levels in A2780, and A2780^DDP^ cells and the activation of the UPR^mt^ pathways were measured with qRT-PCR. Cells (5 × 10^5^/well) were seeded in 6-well plates (Corning), incubated for 24 h then transfected with nanoparticle-siRNA complexes for 48 h. Next, the media was removed, and the cells were lysed with 1 mL TRI Reagent (Sigma-Aldrich) for 10 min. The cell lysate was mixed with chloroform (200 µL) and then centrifuged at 12,000 × *g* for 15 min at 4 °C. The supernatant above the phenol-chloroform fraction was removed, and 2-propanol (500 µL) was added and centrifuged at 12,000 × *g* for 10 min at 4 °C. Next, the supernatant was discarded, and the pellet was washed with 75% ethanol (1 mL) and centrifuged at 7500 rpm for 5 min at 40 °C. The RNA-containing pellet was air-dried, re-suspended in DEPC water (20 µL), and then incubated for 15 min at 56 °C. The RNA concentration was measured with Nanodrop 2000C (Thermo-Fisher Scientific) using the Nanodrop 2000C v.1.4.1 software (Thermo-Fisher Scientific). A GoScript^TM^ Reverse Transcription Kit (Promega Corporation, Madison, WI, USA) was used for cDNA synthesis. Total RNA (500 ng) was annealed with Oligo(dT15) primers at 70 °C for 5 min, then cooled immediately in ice water for 5 min and centrifuged for 10 s. *SOD1* mRNA level was quantified using the (forward) 5′-ATCCTCTATCCAGAAAACACGG and 5′-GCGTTTCCTGTCTTTGTACTTT (reverse), while β-actin housekeeping gene with 5′-CACCATTGGCAATGAGCGGTTCC-3′ (forward) and 5′-GTAGTTTCGTGGATGCCACAGG-3′ (reverse) primer sequences (Sangon Biotech, Shanghai, China) was used as an internal reference. The samples were mixed with 4.0 µL GoScript™ 5X Reaction Buffer (Promega), 3 nM MgCl_2_ (3 µL), 1 µl GoScript™ Reverse Transcriptase, 1 µL PCR Nucleotide Mix (0.5 mM each dNTP), and nuclease-free water (6 μL). The samples were annealed at 25 °C for 5 min, extended at 42 °C for 1 h then incubated at 70 °C for 15 min. The cDNA samples were diluted 10-fold and combined with the GoTaq qPCR Master Mix kit (Promega). A final reaction volume was 20 µL consisting of 2X GoTaq qPCR master mix (10 µl), 10 nM reverse and forward *SOD1* and β-actin primers (1 µL each), cDNA sample (2 µL), and nuclease-free water (6 µL). The PCR cycling parameters were as follows: activation (95 °C, 1 cycle, 2 min), denaturation (95 °C, 15 s), annealing (40 cycles), and extension at 95 °C (1 min). The pentuplet samples were run under SYBR Green setting with an Applied Biosystems^TM^ Quantstudio-5 (Thermo-Fisher Scientific) qRT-PCR machine. The results were analyzed with the Quantstudio-5 Design and Analysis v1.4.3 (Thermo-Fisher Scientific) software using the ∆∆Ct method [33].

#### Western blot (WB)

The baseline protein expression levels of *SOD1*, the knockdown efficiencies of GO^PEI^ and GO^PEI^-mPEG complexed siSOD1 (1, 5, 10, 15, 20, 25, and 30 nM, respectively) at various w/w ratios (0.2:1 to 10:1), and the effect of GO^PEI^ (1–8 µg/mL) treatment alone in A2780 and A2780^DDP^ cells were quantified with WB. Three *SOD1*-specific siRNAs were tested for their knockdown efficiencies: siSOD1-homo-D-333 with sense 5′-CCUCACUUUAAUCCUCUAUTT-3′ and antisense 5′-AUAGAGGAUUAAAGUGAGGTT-3′ sequences, siSOD1-homo-D2757 with sense 5′-CGACGAAGGCCGUGUGCGCTT-3′ and antisense 5′-UCGCACACGGCCUUCGUCGTT-3′ sequences, siSOD1-homo-D2861 with sense 5′-CCCUUAACUCAUCUGUUACTT-3′ and antisense 5′-UUAACAGAUGAGUUAAGGGTT-3′ sequences. All three siRNAs were acquired from Suzhou GenePharma (Suzhou, China). The cells (3 × 10^5^/well) were plated in six-well plates (Corning) and incubated for 24 h in 5% CO_2_ at 37 °C prior to transfection with siSOD1 complexed nanoparticle samples or nanoparticles only using the same method as for FACS. After 48 h, the media was removed, and the plates were washed twice with ice-cold PBS. For protein extraction, 1 X RIPA buffer (200 µL) (150 mM NaCl, 20 mM Tris-HCl, pH 7.5, 1 mM EGTA, 1 mM Na_2_EDTA, 2.5 mM sodium pyrophosphate, 1% NP-40, 1% sodium deoxycholate, 1 mM Na_3_VO_4_, 1 mM β-glycerophosphate, and 1 µg/mL leupeptin) supplemented with 1X protease inhibitor cocktail (Roche, Basel, Switzerland) in a 1:25 v/v ratio. After determining the protein concentration with a BCA Protein Assay kit (BeyoTime, Shanghai, China), the samples were transferred to a 95 °C heat block for 10 min. Next, the respective protein samples (20 µg) were loaded into 10% polyacrylamide 12 or 15-well precast gels (GenScript, NJ, USA) and ran for 90 min at 120 V in Tris-MOPS-SDS running buffer. The gel was transferred with an eBlot™ L1 Fast Wet Transfer System (GenScript) onto a PVDF membrane, then incubated in blocking buffer (Beyotime, Shanghai, China) for 1 h at RT. The membrane was incubated with mouse anti-human *SOD1* (15.9 kDa) monoclonal antibody (Cat# 67480-1-Ig, 1:1000, ProteinTech, Rosemont, IL, USA) and with mouse β-actin (42 kDa) monoclonal antibody (1:2000, Cat# 66009-1-Ig, ProteinTech) overnight at 40 °C. Next, the membrane was washed with 1X TBST thrice for 5 min and incubated with donkey anti-mouse IgG (H + L) IRDye® 800CW (LICOR Biosciences, NE, USA) polyclonal antibody at RT for 1 h, then washed with 1X TBST thrice for 5 min each. Protein bands were displayed by an Odyssey Imaging System (LI-COR Biosciences) and analyzed by the Li-Cor Odyssey 3.0.29 (LI-COR Biosciences) and Image-J (NIH) software.

#### MTT assay

The cisplatin sensitivity (IC_50_) of A2780 and A2780^DDP^ cells in a range of 2–28 µg/mL cisplatin (Sigma-Aldrich) concentration, the cytotoxicity of graphene-based nanocarriers, and the cisplatin chemo-sensitizing effect of nanoparticle complexed siSOD1 (D-333) at different concentrations (1–300 nM), and w/w ratios (0.05:1–10:1) upon cisplatin treatment were first determined with MTT assay before confirming it with clonogenic assay. A2780 and A2780^DDP^ cells (3 × 10^3^ cells/well) in 100 µL complete media were seeded into 96-well plates and incubated for 24 h. Next, cells were treated with the respective drug compound, nanoparticle, or both in serum-free media (50 µL per well) and incubated in 5% CO_2_ at 37 °C for different durations (48–72 h). Next, 15 µL of 5 mg/ml MTT [3-(4,5-Dimethylthiazol-2-yl)-2,5-diphenyltetrazolium bromide] (Sigma-Aldrich) solution was added to each well. The plates were transferred onto a plate shaker, shaken for 5 min at 600 rpm, and then incubated in 5% CO_2_ at 37 °C for 5 h. The formazan crystals were dissolved by adding 135 µL of 10% acidified SDS (Thermo-Fisher Scientific) and further shaken at 500 rpm for 15 min. The absorbance was recorded at 570 nm, and the results (9–18 replicates) were compared to a blank (media only), negative control (cells only), and positive death control (cells treated with Triton X-100 (1%) and analyzed by Varioskan LUX, Skanit Re v.4.1 (Thermo-Fisher Scientific) software. The relative cell viability was calculated as follows:

Cell viability (%) = (OD test sample – OD blank - OD positive control) / (OD negative control – OD blank - OD positive control) × 100%.

#### Population Doubling

To evaluate the effect of *SOD1* knockdown on the proliferation rate, A2780, A2780^DDP^ cells (3 × 10^3^/well) in 100 µL, complete media were seeded into 96-well plates and incubated for 24 h. Next, cells were treated with the respective drug compound, siRNA-nanoparticles, or both in serum-free media (50 µl per well) and incubated in 5% CO_2_ at 37 °C for 4 days. From day 0 to day 4, one 96-well plate was treated with MTT solution, and the absorbance readings were taken at 24 h, 48 h, 72 h, and 96 h post-seeding, respectively. The total normalized absorbance values of the respective plates were used to plot a cell growth curve. Population doubling-time calculations: Population doubling times were calculated using the slope of the angle of the linear regression analysis of the 5 time points and confirmed with the Doubling Time Software v1.0.10 (http://www.doubling-time.com).

#### Clonogenic Assay

The cisplatin sensitivity and chemo-sensitizing effects of GO^PEI^-siSOD1(D-333) and GO^PEI^-mPEG-siSOD1(D-333) were determined with a clonogenic cell assay. A2780 and A2780^DDP^ cells (~1 × 10^3^ cells/well) were seeded in 6-well plates and then incubated overnight with 5% CO_2_ at 37 °C. Next, the different drug compounds, siRNA-nanoparticles, or both dissolved in RPMI (1 mL) media and added to the cells, then following 2–5 h of incubation, depending on the respective treatment, the media was replaced with full media, and the plates were incubated for 10–12 days. The colonies were fixed with methanol (25% v/v) for 1 min, stained with crystal violet (0.05% w/v) for 5 min and washed under running tap water. Colonies (min >50 cells) were counted manually, and the relative cell viability of the treatment groups was expressed relative to the control group.

### In vivo study

#### Ethical approval and animal handling

Animal study protocols (ASP) were approved by the Institutional Animal Care and Use Committee (IACUC) of Suzhou GenePharma (Suzhou, China), and all in vivo experiments were conducted according to IAUCC guidelines. The animals used in this study were housed in the facility of Suzhou GenePharma, an AAALAC internationally accredited research centre. All in vivo experiments were conducted according to the Guide for the Care and Use of Laboratory Animals. Female Balb/c 6–8 weeks old nude mice were exposed to 12 h light/dark cycles, kept in specific pathogen-free conditions in filter-topped cages, and fed with standard rodent chow plus water ad libitum. In this study, mice were sacrificed when any tumour volume reached more than 2000 mm^3^, upon any visible distress expressed by the animals, or more than a 20% decrease in body weight.

#### Preparation of ovarian cancer cells

A2780 cisplatin-sensitive and A2780^DDP^ cisplatin-resistant ovarian cancer cells limited to maximum 10 passages were grown in T-75 flasks and harvested following sterile techniques in a biological safety cabinet in the logarithmic growth phase at 60–70% confluency. The full cell culture media (DMEM supplemented with 10% FBS) was aspirated and washed thrice with PBS (37 °C). Next, trypsin/EDTA (4 mL) was added, and the plates were incubated at 37 °C in 5% CO_2_ for 7 min. The cells were dislodged by gentle plate tapping, and the detached cells were visualized under an inverted microscope. Complete cell culture medium (10 mL) was added to inactivate the trypsin solution, and a single cell suspension was obtained by vigorous mixing. The live cells were counted by mixing 100 μL of the cell suspension with 100 μL of trypan blue and counted in a hemocytometer. The cell number was confirmed by combining 100 μL of the cell suspension in 9900 μL of CASY TT Buffer and counted with the CASY TT automated cell counter (OMNI Life Science GmbH & Co. KG, Bermen, Germany). Next, the single cell suspension (13.8 mL) was transferred to a centrifuge tube (15 mL) and centrifuged at 1000 rpm for 3 min at RT. The supernatant was discarded, and the cells were reconstituted in serum-free media at concentrations of 1 × 10^6^, 5 × 10^6^, or 1 × 10^7^ cells mixed with 50% matrigel per mL as per the experimental design for subcutaneous tumour inoculation experiments. The cells were inoculated in the mice subcutaneously within 30 min of preparation.

#### Nanoparticle hemocompatibility assay

To obtain the red blood cell (RBC) fraction, anticoagulated fresh mouse blood (5 mL) was centrifuged for 10 min at 2000 × *g*, then washed thrice with PBS. Next, 2% (v/v) RBC suspension buffer was prepared in PBS mixed with 5 concentrations of naked siSCR, GO-siSCR, GO^PEI^-siSCR, GO^PEI^-mPEG-siSCR, and Lipo^2000^-siSCR in a total volume of 100 μL added to 900 μL of 2% RBC suspension. The samples were incubated at 37 °C for 1 h, centrifuged for 10 min at 3000 × *g*, and the absorbance was measured at 540 nm using a SpectraMax i3 plate reader.

#### In vivo tumour therapeutic efficacy study of GO^PEI^-mPEG-siSOD1

To evaluate the therapeutic effect of SOD1 knockdown in vivo, Balb/c nude xenograft mice were injected with cisplatin, naked siSOD1(D-2861)+cisplatin, GO^PEI^-mPEG-siSCR (control)+cisplatin, and GO^PEI^-mPEG-siSOD1(D-2861)+cisplatin (*n* = 3), respectively, at 0.5 mg/kg siRNA-nanoparticles dose. During a 14-day long therapeutic window, mice received 6 doses of respective siSCR or siSOD1(D-2861)-based treatments with or without cisplatin treatment. Tumour sizes were measured by caliper every 2–3 days.

#### In vivo toxicity and blood biochemistry testing

The potential acute in vivo toxicity of the nanoparticles was evaluated with blood biochemistry testing. Balb/c nude mice were injected with siSCR, GO-siSCR, GO^PEI^-siSCR, GO^PEI^-mPEG-siSCR (*n* = 3) at a 0.5 mg/kg siRNA dose. Blood samples were collected 8 h after the drug administration. Whole blood samples without anticoagulants were centrifuged at 3000 × *g* at 4 °C for 10 min to obtain the serum fraction. Haematological testing was performed using an automatic haematology analyzer (MEK6400, Nihon Kohden, Japan) for serum alanine aminotransferase (ALT), aspartate aminotransferase (AST), alkaline phosphatase (ALP), total protein (TP), albumin (ALB), creatinine (CREA), uric acid (UA), blood urea nitrogen (BUN) lactate dehydrogenase (LDH) and amylase (AMY).

### Statistical analysis

Unless stated otherwise, all experiments were carried out in triplicates (*n* = 3). The in vitro numerical data were analyzed for statistical significance with Student’s two-tailed paired and unpaired t-tests and were expressed as mean ± SD using GraphPad Prism 7.04 (GraphPad Software, San Diego, CA. USA). In the study design, *p* < 0.05 was considered statistically significant and was denoted as *, **, ***, and **** for less than 0.05, 0.01, 0.001 and 0.0001, respectively.

## Results

### Graphene nanoparticle preparation

**Fig. 1:**
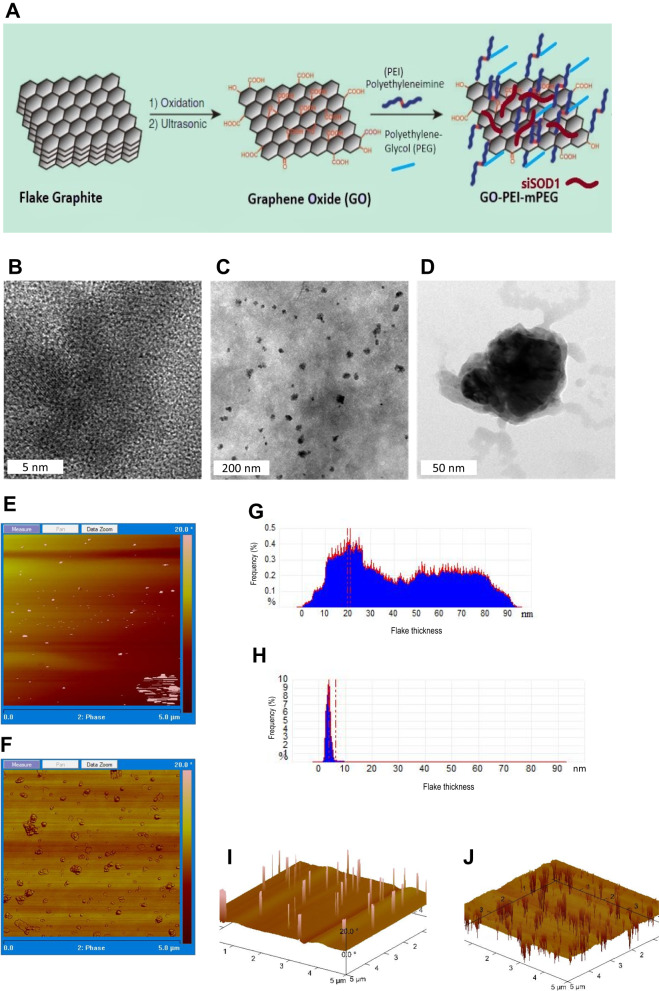
Overview of nanoparticle synthesis and morphological characterization. **A** Schematic representation of GO^PEI^-mPEG preparation. mPEG was directly conjugated to PEI instead of graphene, which created a tunable system to control the ζ-potential of the nanoparticle. Linear methyl polyethylene glycol (mPEG) was conjugated to GO^PEI^ through EDC chemistry, and siRNA molecules were allowed to bind GO^PEI^-mPEG electrostatically. **B**, **C** High-resolution TEM images of the surface morphological characterization of GO at different scales (5 nm, 200 nm, and 50 nm, respectively) showing a well-dispersed homogenous nanoparticle solution with varying sizes and dimensions. **D** Single GO^PEI^ nanoparticle with rough and wrinkle surface characteristics. AFM analysis of GO and GO^PEI^ surface morphology (**E**, **F**), lateral thickness (**G**, **H**), and topographical 3-D images (**I**, **J**) respectively. AFM analysis showed an even distribution of GO flakes with lateral dimensions in the 0.05–0.4 μm range and thickness of 1–2 layers of GO and GO^PEI^.

Graphene-oxide (GO) was prepared according to a previous study (Fig. 1A) [32]. Subsequently, polyethyleneimine (PEI) was conjugated onto the GO sheets using EDC chemistry, yielding a GO-PEI (GO^PEI^) nanocarrier. Further, to increase biocompatibility and decrease surface charge, GO^PEI^ was directly PEGylated on the primary amine groups of PEI (GO^PEI^-mPEG), allowing for a tunable surface charge configuration. TEM images of GO and GO^PEI^ showed distinct hexagonal lattice structure of GO (Fig. 1B) and a homogenous, well-dispersed nanoparticle distribution exhibiting rough, wrinkled morphological domains of graphene sheets (Fig. 1C, D). A high-resolution image of GO^PEI^ revealed darker sheet intensities relative to GO due to PEI functionalization (Fig. 1D). AFM imaging revealed two-dimensional sheet-like morphology with highly variable particle thickness and a lateral size ranging from 0.05 to 0.40 μm. (Fig. 1E, F) Following PEI conjugation, the sheet thickness of GO^PEI^ increased by ~3–4 nm relative to GO. (Fig. 1G, H) The latter was further confirmed with z-section analysis showing darker sheet intensities on 3D topographic analysis of the GO^PEI^ sample attributed to increased flake thickness due to PEI functionalization (Fig. 1I, J).

### Graphene nanoparticle characterization

**Fig. 2:**
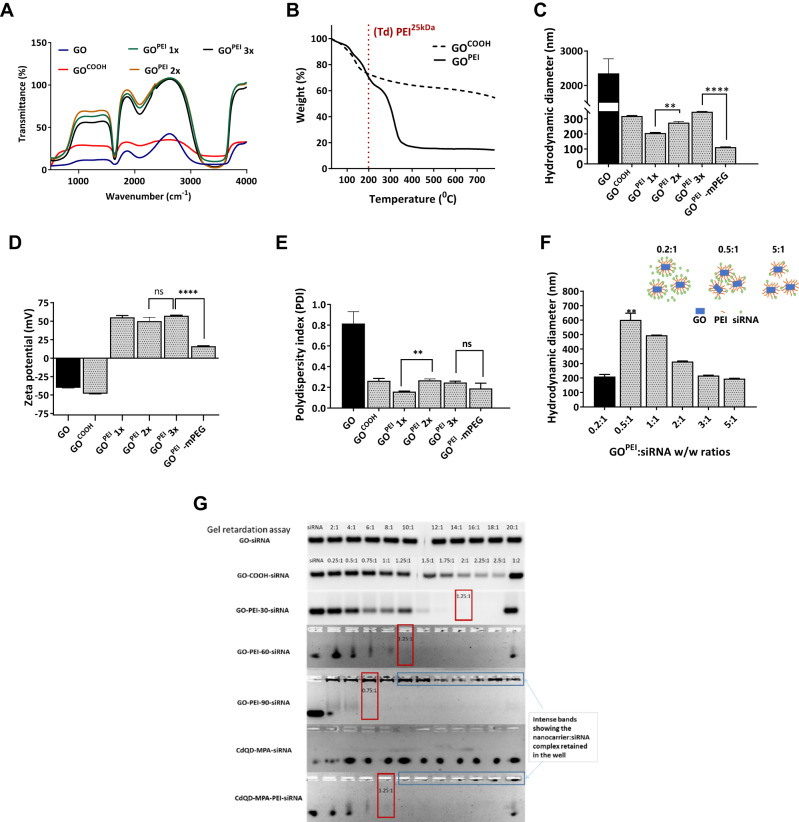
Overview of nanoparticle structural and functional characterization. **A** FT-IR spectra of GO and increasing concentrations of cationic polymer functionalized GO^PEI1x^, GO^PEI2x^, GO^PEI3x^. Conjugation of the carboxyl group of GO and the amino group of PEI was confirmed by FT-IR spectroscopy by measuring the characteristic peak of the amide bonds at 1630–1695 cm^−1^. **B** TGA spectra of GO and GO^PEI^ was measured in the temperature range of 25-800 ˚C. The decomposition of components of the nanoformulation brought about by the mass loss at increasing temperatures was used to estimate the PEI content in GO^PEI^. (**C**–**E**) DLS analyses of (**C**) hydrodynamic size, (**D**) ζ-potential, and (**E**) PDI of the nanoparticles GO, GO^PEI1x^, GO^PEI2x^, GO^PEI3x^, and GO^PEI^-mPEG. The particle sizes were 2354.6 ± 423, 318.7 ± 2.7, 206.0 ± 2.7, 274.8 ± 7.2, 347.4 ± 1.2, and 112 ± 1.2 nm, respectively. The ζ-potential were −40.4 ± 0.4, −48.1 ± 0.4, 55.2 ± 2.4, 50.1 ± 5.1, 57.3 ± 0.8, and 16.1 ± 0.6 mV, respectively. **F** In vitro evaluation of GO^PEI^-siSCR aggregation at different ratios. A DLS study measuring the hydrodynamic size of GO^PEI^-siSCR at different w/w ratios was carried out (*n* = 3) at the following ratios: 0.2:1, 0.5:1, 1:1, 2:1, 3:1, and 5:1. **G** Agarose gel retardation assay of the siSCR with GO and Cadmium quantum dots (CdQD) nanoparticles, respectively. GO and CdQD at various weight ratios of 2:1, 4:1, 6:1, 8:1, 10:1, 12:1, 14:1, 16:1, 18:1 and 20:1. Lipo^2000^, GO^PEI1x^, GO^PEI2x^, GO^PEI3x^ and GO^PEI^-mPEG used w/w ratios of 0.25:1, 0.5:1, 0.75:1, 1:1, 1.25:1, 1.5:1, 1.75:1, 2:1, 2.25:1 and 2.5:1. Naked siSCR was used as negative control. The red frame represents the best complexation w/w proportion. All values are expressed as mean ± SD. ns- not significant and **p < 0.01 as analyzed using a two-tailed unpaired *t*-test.

Next, we confirmed whether the conjugation of -COOH groups of GO to the primary amines of PEI occurred using FT-IR spectroscopy (Fig. 2A). A characteristic peak of amide bonding was observed at 1630–1695 cm^−1^ of GO^PEI^ but was absent in GO samples [34]. A broad peak at 1705–1730 cm^−1^, characteristic of carboxyl groups, and further peaks at 1200–1300 cm^−1^ and 3200–3500 cm^−1^ corresponding to C=O and –OH groups were appreciable of GO. We further measured the composition of GO^PEI^ using TGA. The thermogravimetric curve of GO^PEI^ (Fig. 2B) showed a biphasic decomposition pattern relative to GO. Both samples exhibited rapid initial weight loss (<150 °C) attributed to remnant water evaporation. However, the mass loss of GO^PEI^ in the 250–350 °C range increased considerably compared to GO due to PEI^25 kDa^ decomposition (T_d_ PEI^25 kDa^ ~ 200 °C). Based on these results we concluded that PEI^25 kDa^ makes up ~70% of the total mass of GO^PEI^. Next, the absorbance spectrum (Fig. S1A) and electrostatic adsorption of siSCR cargo onto GO^PEI^ (Fig. S1B) were evaluated with UV–Vis spectroscopy. The siSCR complexed GO^PEI^ showed higher and superimposed (GO^PEI^ + siSCR) absorbance spectrum compared to their individual peaks indicating electrostatic complexation of the siSCR cargo with the GO^PEI^ carrier (Fig. S1B). In addition to conventional TEM- and AFM-based graphene characterisation, the nanoparticle size distribution was periodically monitored before in vitro assays for potential aggregation using DLS to lower batch-to-batch variations and to increase experimental reproducibility. Next, for our reference, we established that the prepared GO had an average size distribution of 2354.6 ± 423 nm (Fig. 2C) and a low ζ-potential of −40.4 ± 0.4 mV (Fig. 2D), showing high size heterogeneity in solution with a PDI of 0.81 ± 0.11 (Fig. 2E). Following the activation of the –COOH groups with NaOH, GO exhibited lower hydrodynamic size distribution (318.7 ± 2.7 nm), and lower ζ-potential (−48.1 ± 0.4 mV), and the carboxylation lowered (*p* < 0.05) the polydispersity of the graphene sheets (0.263 ± 0.04). Different batches of GO^PEI^ corresponding to various PEI concentrations showed increasing hydrodynamic sizes (*p* < 0.01) (206.0 ± 2.7 nm, 274.8 ± 7.2 nm and 347.4 ± 1.2 nm) and stable ζ-potential between +50–60 mV (55.2 ± 2.4 mV, 50.1 ± 5.1 mV and 57.3 ± 0.8 mV) and PDI (0.159 ± 0.01, 0.267 ± 0.01 and 0.247 ± 0.01) (Fig. 2C–E). GO^PEI^-mPEG exhibited similar physicochemical properties as GO^PEI^ with a reduced lateral size of 129.3 ± 0.1 nm (*p* < 0.0001), and decreased ζ-potential (16.8 mV, *p* < 0.0001), and no significant change (*p* > 0.05) in PDI was appreciable following mPEG conjugation. Further, we confirmed that the hydrodynamic diameter of the graphene-siRNA polyplexes changed by modulating the GO^PEI^-mPEG to siRNA weight-to-weight (w/w) complexation ratios (Fig. 2F). Finally, the loading capacity, electrostatic adsorption, and retention of siSCR of the nanoparticles were assessed by agarose gel retardation assay (Fig. 2G). Relative to the control naked siSCR bands, the nanoparticle complexed siSCR groups showed lower band intensities indicating partial or total siSCR loading and retention at different w/w ratios. GO^PEI^ nanoparticles containing increased amounts of PEI fully complexed siSCR at w/w ratios of 2:1, 1.25:1, and 0.75:1, respectively, while GO^PEI^-mPEG fully complexed the siSCR cargo at 1.25:1 w/w ratio. However, non-functionalized GO failed to completely retain siSCR, as evidenced by the presence of free siSCR bands at high w/w ratios. However, GO showed partial retention of siSCR, which is consistent with prior literature [35]. These findings were further validated in the last well of each nanoparticle group, where the nanoparticle to siSCR complexation ratio was 1:2, and the band intensity was comparable to the 0.5:1 group.

### Evaluation of in vitro biocompatibility

**Fig. 3:**
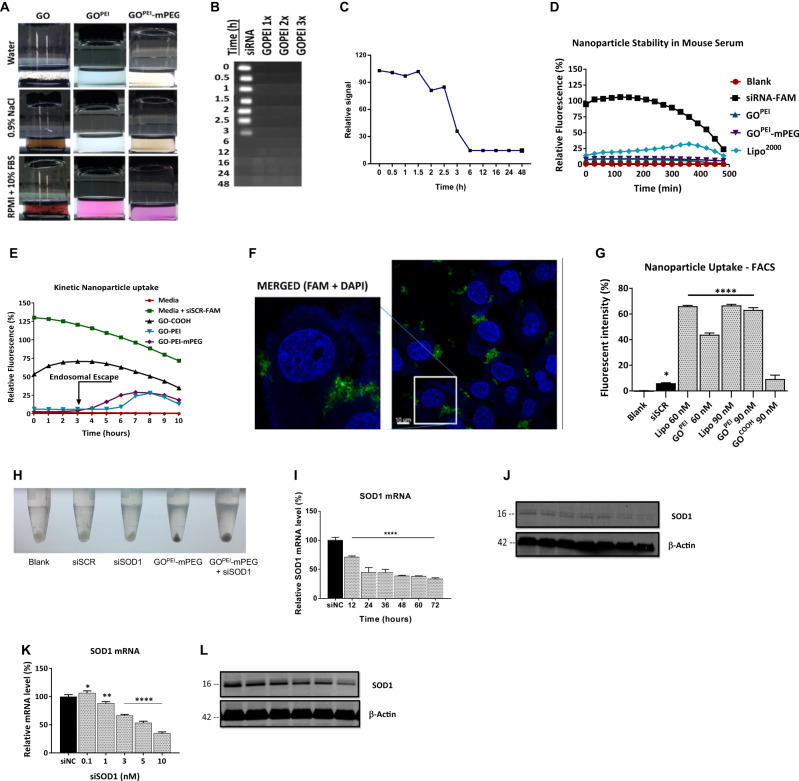
Nanoparticle behaviour in biological solutions. **A** Colloidal stability of GO, GO^PEI^, and GO^PEI^-mPEG were assessed in DIW, 0.9% NaCl, and RPMI + 10% FBS, 1 h after treatment. Nanoparticle aggregation was observed in all three biological solutions containing non-functionalized GO. **B**, **C** Gel-electrophoresis-based serum stability was carried out in GO^PEI1x^, GO^PEI2x^, and GO^PEI3x^. The susceptibility of siRNA to RNase degradation in serum was assessed for 48 h. Naked siSCR was used as a control. **D** Kinetic study of nanocarrier-siRNA serum stability was measured using mouse serum in siSCR^FAM^ conjugated GO^PEI^, GO^PEI^-mPEG, and Lipo^2000^ relative to control naked siSCR^FAM^. The fluorescence of siSCR^FAM^ was measured over a time of up to 8 h. **E** Kinetic study of nanoparticle behaviour was conducted in vitro using siSCR^FAM^ complexed GO, GO^PEI^ and GO^PEI^-mPEG. The degree of quenching induced by GO^PEI^ upon binding siSCR^FAM^ was measured by a Varioskan fluorescent plate reader. A 10 h time-course study of the live cell uptake of the nanoparticles was measured by recording their fluorescent emission. Samples containing only cells and RPMI media were used as negative controls. **F** Confocal laser scanning images showing the cellular uptake of GO^PEI^- siSCR^FAM^. Merged fluorescence images of cells treated with GO^PEI^- siSCR^FAM^ with a magnified image of a single A2780 cell showing successful transfection of GO^PEI^ as aggregations were compared with Lipo^2000^ as the positive control, and naked siSCR^FAM^ was used as a negative control. **G** Quantitative assessment of nanoparticle: siRNA cellular uptake GO^PEI^-siRNA transfection efficiency was analyzed using flow cytometry. Two siSCR^FAM^ concentrations of 60 nM and 90 nM complexed with Lipo^2000^ served as positive control. **H** Image showing intracellular graphene accumulation in cell pellets following three washing steps and centrifugation prior to protein isolation. **I**, **J** Time-course study showing *SOD1* mRNA and protein levels following *SOD1* knockdown. **K**, **L** The effect of concentration dependent *SOD1* knockdown on protein levels was determined by Western blot and mRNA expression levels were measured using RT-qPCR. All values are expressed as mean ± SD. ns- not significant, *p < 0.05, **p < 0.01, and ****p < 0.0001 as analyzed using two-tailed unpaired *t*-test.

The aggregation potential of nanoparticles in biological solutions was observed and measured over 1 h, 24 h, 1, 3, and 6 months (Fig. 3A). We found that GO immediately precipitated and formed visible aggregates upon mixing with 0.9% NaCl, PBS, RPMI (10% FBS), and FBS (50%) solutions that persisted during the observation period (6 months) (Fig. S1C). GO^PEI^, however, did not show any observable aggregation and remained a homogenous solution even after 1 month of incubation in DIW, PBS, RPMI (10% FBS), FBS (50%), NaCl (0.9%) or RPMI (10% FBS, pH: 6.4) at RT that aimed to simulate the microenvironment of solid tumours. Further, the serum stability of GO^PEI^ complexed siSCR evaluated against RNase degradation showed adequate complexation and protection of the siRNA cargo by the prepared nanoparticles in high serum-containing solutions (Fig. 3B, C). While naked siSCR completely degraded and its band disappeared after 6 h, GO^PEI^ nanoparticles showed no bands after complexation with siSCR throughout the study even after 48 h in 50% FBS-containing solution, which simulated human plasma compared to negative control samples (Fig. S1D).

The agarose gel-based serum stability study results were further validated with a fluorescent kinetic study using mouse serum (Fig. 3D). The emitted fluorescence of nanoparticles complexed with siSCR^FAM^ samples were recorded for 8 h. The negative control naked siSCR^FAM^ group showed a rapid drop in fluorescence after 3 h and completely disappeared after 6 h, attributed mainly to degradation by RNAses of the mouse serum, which is in line with the results of the gel-based assay stability study. The GO^PEI^ and GO^PEI^-mPEG complexed siSCR^FAM^ samples steadily showed low fluorescence during the 8 h long study, indicating that a negligible amount of siSCR^FAM^ was released from the nanoparticles during incubation compared to the positive control Lipo^2000^ group. Overall, we established that the prepared GO^PEI^ and GO^PEI^-mPEG nanoparticles could provide sufficient protection for the siRNA cargo for a minimum of 4 h and were deemed suitable for further in vivo experiments.

Next, we evaluated the kinetic cellular uptake of the nanoparticles in a kinetic in vitro assay (Fig. 3E). During a 10 h time-course study using live cells, we observed increased siSCR^FAM^ fluorescence after 6 h for GO^PEI^ and GO^PEI^-mPEG nanoparticles but not for naked siSCR^FAM^. We attributed this increase in siSCR^FAM^ fluorescence to the endosomal escape of the nanoparticles mediated by the proton sponge effect of PEI^25 kDa^ in the acidic lysosomal environment, since under physiological and buffered pH conditions, our serum stability assay showed (Fig. S1D), that GO^PEI^ managed to fully complex and prevent releasing the siSCR cargo for an extended period of 48 h [36]. The qualitative intracellular uptake of GO^PEI^-siSCR^FAM^ complexes was also investigated by laser confocal microscopy (Fig. 3F, Fig. S1E). A2780 cells, in addition to control groups, were transfected naked siSCR, GO^PEI^, Lipo^2000^, GO^PEI^-siSCR^FAM^, and Lipo^2000^-siSCR^FAM^ to measure the intracellular fluorescence following successful siRNA cargo delivery by GO^PEI^ to the cells. GO^PEI^-siSCR^FAM^ and Lipo^2000^-siSCR^FAM^ showed evident green fluorescence in the cytoplasm, while for naked siSCR^FAM^ no fluorescence was detectable. Similarly, cells treated with media or nanoparticles only (GO^PEI^ or Lipo^2000^) showed no fluorescence. Finally, the quantitative cellular uptake of nanoparticle complexed siSCR^FAM^ polyplexes were measured with flow cytometry (Fig. 3G and S1F). Naked siSCR^FAM^ was partially taken up by A2780 cells, and unfunctionalized GO showed minimal siRNA delivery capability. In contrast, GO^PEI^ complexed siSCR^FAM^ (60, 90 nM) at a 3:1 w/w ratio showed significantly increased fluorescence (43.89% and 63.21%, *p* < 0.0001) comparable to the positive control Lipo^2000^ group with 66.00% and 66.20% using 60 and 90 nM siSCR, respectively. In addition, cellular uptake of the graphene-based nanoparticles was evident during protein isolation, as cell pellets showed evident dark discolouration attributed to the presence of intracellular graphene nanoparticles (Fig. 3H). GO^PEI^ complexed with 90 nM siSCR^FAM^ showed the highest fluorescence and thus was selected for further experiments. Finally, we determined that GO^PEI^-mPEG-siSOD1 can efficiently silence *SOD1* expression on both mRNA (Fig. 3I) and protein (Fig. 3J) levels. In addition, time-course evaluation of *SOD1* silencing showed potent downregulation of mRNA (Fig. 3K) and protein levels (Fig. 3L) after 24 and 72 h, respectively. We have also used the other three different cell lines, HeLa, A549, and Hep3B, to confirm the siRNA’s ability to downregulate *SOD1* expression at the protein level (data not shown).

### Evaluation of in vitro characteristics in cell-based assays

**Fig. 4:**
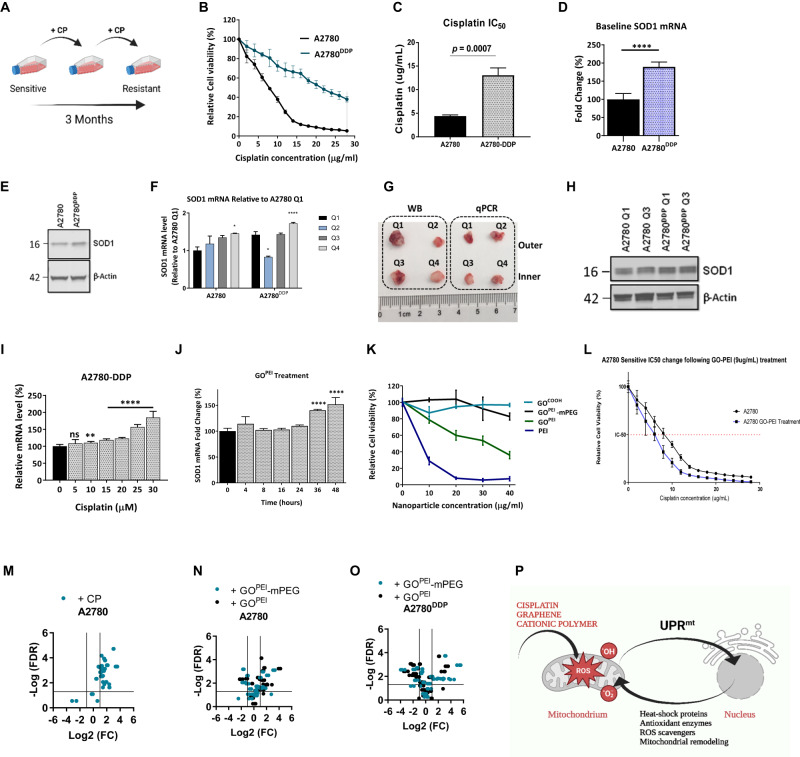
In vitro nanoparticle toxicity. **A** Schematic diagram of the in vitro induction of acquired platinum resistance. **B** Baseline cisplatin sensitivity of A2780 and A2780^DDP^ cell lines. **C** The IC_50_ of A2780 and A2780^DDP^ were 4.71 ± 0.26 and 13.95 ± 1.18 µg/ml, respectively. **D** Bar graph showing normalized baseline *SOD1* mRNA levels (*n* = 6). **E** Western blot showing the SOD1 protein levels in A2780 and A2780^DDP^ cell lines in vivo in Q1 and Q3. **F** Bar graph showing normalized baseline *SOD1* mRNA (*n* = 3). **G** Schematic illustration of xenograft tumour sample preparation for immunoblotting and qRT-PCR. **H** Western blot showing the SOD1 protein levels in A2780 and A2780^DDP^ cell-derived xenograft tumour tissue samples. **I** Cisplatin treatment of A2780^DDP^ cells (*n* = 6) induced *SOD1* mRNA overexpression in a concentration-dependent manner measured by RT-qPCR. **J** Time-course evaluation *SOD1* mRNA induction by GO^PEI^ treatment in A2780^DDP^ cells (*n* = 6) measured by qRT-PCR. **K** Cytotoxicity of GO, PEI, GO^PEI^ and GO^PEI^-mPEG were determined at the following concentrations: 2, 4, 6, 8, 10, 12, 14, 16, 18, 20, 25, 30, 35 and 40 µg/mL, respectively. **L** MTT assay showing relative cell viability after A2780 cells were treated with 9 µg/mL of GO^PEI^ for 48 h followed by cisplatin treatment at 14 different concentrations (2, 4, 6, 8, 10, 12, 14, 16, 18, 20, 22, 24, 26 and 28 µg/mL respectively). IC_50_ values were compared with that of A2780 cells (*n* = 6). **M** Volcano plot showing the transcriptional activation of the mitochondrial unfolded protein response by 15 µM cisplatin in A2780 cell line. **N**, **O** GO^PEI^ and GO^PEI^-mPEG treatment-induced differential in vitro activation of the UPR^mt^ in (**N**) A2780 and (**O**) A2780^DDP^ cell lines. **P** Schematic diagram illustrating UPR^mt^ activation by cisplatin, graphene and cationic polymers leading to mitochondrial dysfunction and subsequent mito-nuclear signalling.

In the present study, to model the development of acquired cisplatin resistance, we first exposed human epithelial A2780 ovarian cancer cells to low-dose cisplatin (1 µM) for 3 months (Fig. 4A). Next, the degree of acquired cisplatin resistance induction was determined in the A2780 and resistant pair of A2780^DDP^ cells (Fig. 4B, C), and we observed a ~3-fold increase in cisplatin IC_50_ with MTT assay (*p* = 0.0007), that we confirmed with clonogenic assay (Fig. S1G, H). To establish causality between acquired cisplatin resistance and *SOD1* overexpression in vitro, we first determined the baseline *SOD1* mRNA and protein levels between the cell lines (Fig. 4D, E). Consistent with our previous findings, A2780^DDP^ exhibited a 1.61-fold increase in *SOD1* mRNA (Fig. 4D) and a 1.3-fold increase in protein levels in vitro (Fig. 4E). The same cell line pair showed consistently increased (*p* > 0.001) *SOD1* mRNA (Fig. 4F) while a notable 1.9-fold increase in protein levels in vivo (Fig. 4G) in four independent tumour quadrants (Fig. 4H). Interestingly, however, when measured in two outers and two inner tumour quadrants, we observed inhomogenous and fluctuating *SOD1* mRNA expression levels between the respective quadrants, that we mechanistically validated on the protein levels in an independent study (unpublished observation). Based on these findings, we concluded that in our model, acquired cisplatin resistance is associated with *SOD1* overexpression both in vitro and in vivo. To establish causality between cisplatin treatment and *SOD1* overexpression, we measured the change of *SOD1* mRNA levels upon a range of 0–30 µM cisplatin treatment. We found that cisplatin treatment elicited significant *SOD1* mRNA overexpression (*p* < 0.0001) (Fig. 4I). In addition, we observed that GO^PEI^ solely (1–8 µg/mL) could significantly (*p* < 0.001) increase *SOD1* mRNA levels above 4 µg/mL attributed to the combined effect of oxidative stress induction by PEI and mechanical shear stress elicited by the GO sheets (Fig. 4J). Next, we evaluated the in vitro cytotoxicity of the nanocarriers and their effect on cell viability using the MTT assay (Fig. 4K). GO treatment showed relatively high cell viability (80%) even at 40 µg/ml concentration. In contrast, free PEI^25 kDa^, as anticipated, showed concentration-dependent toxicity and caused complete cell death (*p* < 0.0001) at or above 20 µg/mL concentration. As per our previous findings, the conjugation of GO with PEI significantly increased cell viability compared to free PEI alone (*p* < 0.0001) [34], while subsequent PEGylation further decreased cellular toxicity. In addition, combinational treatment with GO^PEI^ (9 µg/mL) with cisplatin (2–28 µg/mL) resulted in a considerable decrease in cisplatin IC_50_ (7.71–6.24 µg/ml) (Fig. 4L) relative to the GO^PEI^ (9 µg/mL) treated control. However, we attributed this chemosensitization effect not to *SOD1* knockdown but rather the toxicity of PEI, which evidently necessitated the PEGylation of the nanoparticle.

Next, we aimed to further understand the potential mechanism governing cisplatin and GO^PEI^ elicited *SOD1* mRNA upregulation and hypothesized the involvement of oxidative stress induction and mitochondrial damage. Cisplatin, GO, and PEI have been reported as causative agents of mitochondrial dysfunction [37]. In the mitochondria, *SOD1* is located only in the intermembrane space. In healthy non-pathologic cells, it neutralizes superoxide anions generated by complex III of the electron transport chain, and it is also part of the executive branch of the UPR^mt^ mediating mitoprotective functions primarily upon mitochondrial oxidative stress [38]. Thus, to explore the relationship between cisplatin or nanoparticle-mediated toxicity and the proposed activation of the UPR^mt^, using a set of UPR^mt^-specific primers, we monitored the transcriptional activation of the respective signalling pathways and found that cisplatin-induced mitochondrial damage can activate the UPR^mt^ (Fig. 4M) in A2780 ovarian cancer cells on the mRNA and protein levels (unpublished observation). Further, we concluded that both GO^PEI^ and GO^PEI^-mPEG had similar effect in the same cell line (Fig. 4N), while PEGylation did not entirely prevent the ROS-mediated activation of the UPR^mt^ signalling. In addition, GO^PEI^ and GO^PEI^-mPEG treatment of A2780^DDP^ cells showed partial UPR^mt^ activation (Fig. 4O), however, at a significantly lower magnitude. Further evaluation of additional mechanistic links between cisplatin or graphene nanoparticle-mediated UPR^mt^ activation is beyond the scope of this study. Although, evidently different compounds can elicit varying degrees of UPR^mt^ activation (Fig. 4P), implying specificity in the underlying mechanism and potential correlation with cisplatin-sensitivity, that merits further investigation in the future. Overall, based on the in vitro study results, we concluded that the prepared GO^PEI^-mPEG nanoparticle has acceptable biosafety properties for subsequent in vivo studies.

### SOD1 knockdown reverses cisplatin resistance in vitro

**Fig. 5:**
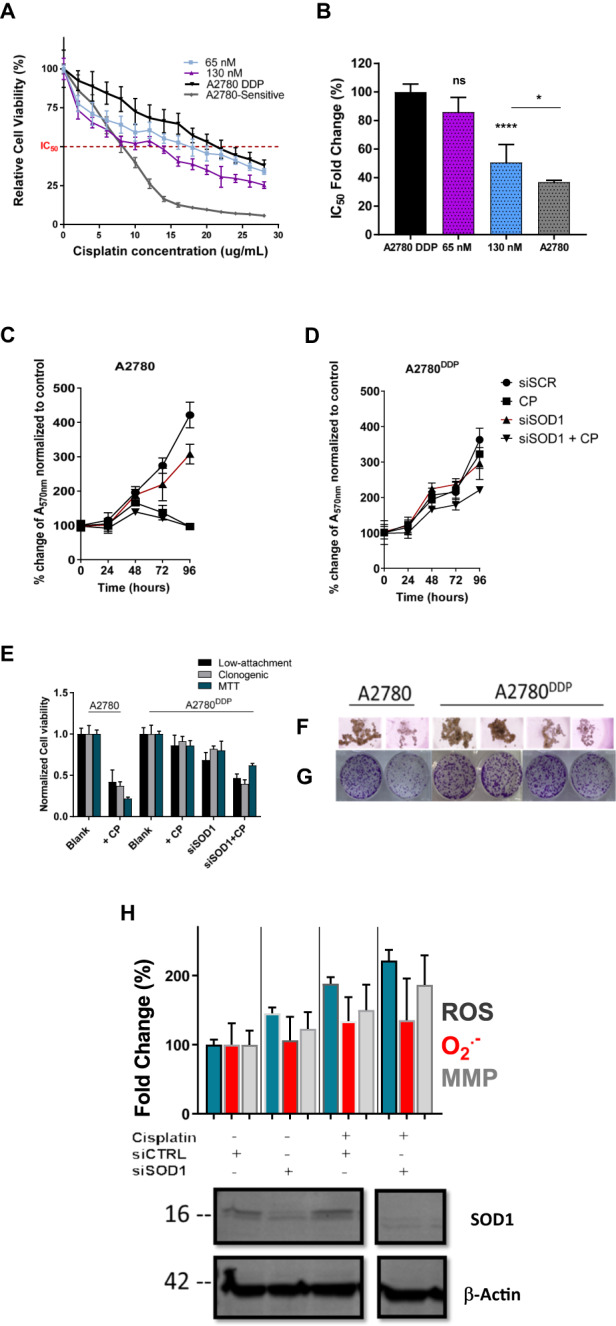
Reversal of cisplatin resistance in vitro. **A** MTT assays showing reversal of cisplatin resistance by GO^PEI^-siSOD1. Relative cell viability of A2780^DDP^ cells treated with 65 and 130 nM of GO^PEI^-siSOD1 at a w/w ratio of 0.2:1 and subsequently with cisplatin (2–28 µg/mL). The graph also shows the relative cell viability of A2780 and A2780^DDP^ cells relative to cisplatin treatment only as a reference (*n* = 18). **B** Change of cisplatin IC_50_ values normalized to that of A2780^DDP^ following treatment. **C** Population doubling curves of the A2780 cell line (0–96 h) under various treatment conditions. **D** Population doubling curves of the A2780^DDP^ cell line (0–96 h) under various treatment conditions. **E** Therapeutic efficacy and antiproliferation effect of GO^PEI^-mPEG-siSOD1 in A2780 and A2780^DDP^ cells evaluated in ultra-low attachment plates, clonogenic and MTT assays. All values have been normalized to their respective untreated cell line control, and presented as mean ± SD (*n* = 3). **F**, **G** Representative images of ultra-low attachment and clonogenic assay cell culture plates. **H** Evaluation of total ROS, superoxide and mitochondrial membrane potential with plate reader under siSCR control and siSOD1 knockdown conditions confirmed with Western blot.

The in vitro chemo-sensitizing effect of GO^PEI^-mPEG-siSOD1 was first evaluated by co-treating A2780^DDP^ cells with siSOD1 (65 and 130 nM, respectively) (Fig. 5A). We observed that GO^PEI^-mPEG-siSOD1 treatment lowered cisplatin IC_50_ values (*p* < 0.0001) from a baseline 18.31 ± 0.53 µg/mL to 7.42 ± 0.79 upon treatment with 130 nM siSOD1 complexed GO^PEI^-mPEG relative to the GO^PEI^-mPEG-siSCR control group (Fig. 5B). Further, we observed that *SOD1* knockdown caused considerable cell growth inhibition, evident from the drop in population doubling times in both examined cell lines (Fig. 5C, D). In addition, the in vitro therapeutic efficiency of *SOD1* knockdown was measured using ultra-low attachment plates, clonogenic assay and MTT assay using a fixed concentration of 15 µM cisplatin. We concluded that under all of these conditions, *SOD1* knockdown sensitized the A2780^DDP^ cells to cisplatin (Fig. 5E–G). Finally, we measured the total ROS, superoxide, and mitochondrial membrane potential using DCHF-DA, MitoSox-Red, and JC-1 fluorescent dyes (Fig. 5H). We found that *SOD1* knockdown alone and in combination with cisplatin treatment resulted in elevated total ROS (*p* < 0.0001) and further caused mitochondrial membrane depolarization (*p* < 0.05). We concluded that *SOD1* knockdown is associated with significant cellular oxidative stress and mitochondrial toxicity, which may contribute to the sensitization of cisplatin in the A2780^DDP^ cisplatin-resistant cells.

### Evaluation of in vivo biodistribution, biosafety, and therapeutic efficacy of graphene-based nanoparticles

**Fig. 6:**
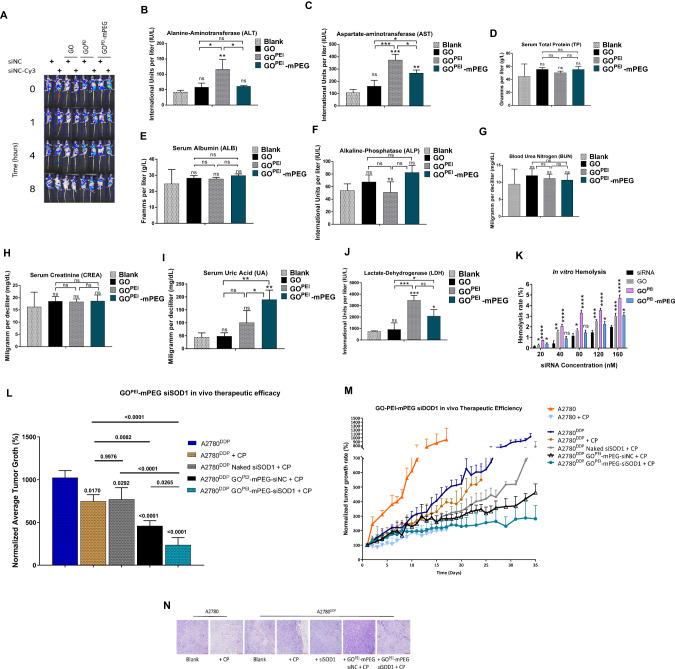
Evaluation of in vivo biodistribution, toxicity, and therapeutic efficiency of graphene-based nanoparticles. **A** In vivo fluorescence imaging and biodistribution siSCR and siSCR^Cy3^ complexed nanoparticle formulations administered via tail vein injection of female BALB/c nude mice bearing A2780^DDP^ subcutaneous xenografts. Whole body fluorescence images were taken at different time points (0, 1, 4, 8 h) following tail vein injection. **B**–**J** In vivo blood biochemistry analysis of (**B**) ALT (**C**) AST (**D**) TP (**E**) ALB (**F**) ALP (**G**) BUN (**H**) CREA (**I**) UA (**J**) LDH of mouse samples following systemic administration of nanoparticles. **K** In vitro hemolysis activity of naked siSCR, GO, GO^PEI^ and GO^PEI^-mPEG nanoparticles. Hemolysis rate was measured following incubation of nanoparticle polyplexes with purified red blood cells in PBS. **L** H&E staining of the heart, kidney, liver, spleen and lungs harvested from mice recieved intravenous naked siSCR, and siSCR^Cy3^ complexed GO, GO^PEI^ and GO^PEI^-mPEG respectively. Images of collected organs from each group was taken at 8 h after tail vein injection. **M** In vivo tumour therapeutic efficiency of GO^PEI^-mPEG-siSOD1. During a 14-day long therapeutic window, mice received 6 doses of respective siSCR or siSOD1-based treatments with or without cisplatin treatment. **N** H&E staining of A2780 and A2780^DDP^ cell-derived tumour cross-sections in their respective treatment groups as in (Fig. 6M) harvested from mice three days after the last injection.

The in vivo pharmacokinetic and pharmacodynamic properties of GO^PEI^-mPEG were evaluated using whole-body fluorescent imaging (Fig. 6A). Using normal saline and naked siSCR^Cy3^ as controls, mice bearing A2780^DDP^ xenografts were administered GO, GO^PEI^, and GO^PEI^-mPEG complexed with siSCR^Cy3^ via the tail vein and the nanoparticle distribution was monitored at different times points (0, 1, 4, and 8 h). The fluorescent intensity of naked siSCR^Cy3^, GO^PEI^, and GO^PEI^-mPEG groups gradually increased during the study. However, no significant change in fluorescence was observed with non-functionalized GO, consistent with previous reports [35, 39]. The recorded fluorescent intensity was comparable between GO^PEI^- siSCR^Cy3^ and GO^PEI^-mPEG-siSCR^Cy3^ groups compared to their respective siSCR control groups. We concluded that GO^PEI^ and GO^PEI^-mPEG could accumulate in tumour tissues and deliver the siSCR^Cy3^ cargo. In addition, these results suggested that PEGylation of GO^PEI^ did not significantly alter the drug loading and delivery capacity of the nanoparticle, a common issue encountered following PEGylation of nanoparticles [40].

In addition, blood biochemistry parameters (Fig. 6B–J) were obtained to monitor acute systemic toxicity following intravenous nanoparticle administration via the tail vein. We found that GO^PEI^-mPEG exhibited lower liver function indices, including ALT (*p* < 0.05) and AST (*p* < 0.05), than GO^PEI^. However, both GO^PEI^ and GO^PEI^-mPEG nanoparticles showed significantly higher levels of AST (*p* < 0.0001 and *p* < 0.001, respectively), while only GO^PEI^ treatment showed ALT elevation (*p* < 0.001) compared to the blank saline control group. Further, only GO^PEI^-mPEG showed UA level elevation (*p* < 0.001), while both nanoparticles significantly raised serum LDH levels (*p* < 0.001 and *p* < 0.01, respectively). We concluded that both nanoparticles elicited toxicity to a certain degree in vivo. Furthermore, PEGylation of GO^PEI^ did not entirely circumvent but still considerably decreased the toxicity induced presumably by GO or the PEI cationic polymer functional group.

Further, in vitro, hemocompatibility testing using whole mouse blood showed that GO^PEI^-complexed siSCR induced a significantly higher (*p* < 0.0001) hemolysis rate relative to naked siSCR and GO controls in a concentration-dependent manner (0–160 nM). This effect was considerably reduced by the PEGylation of GO^PEI^ (*p* < 0.01) (Fig. 6K). It has been reported previously that cationic gene-drug carriers would cause blood toxicity by hemolysis and organic damage after administration in vivo [41]. As both GO and PEI have been known to induce hemolysis, we concluded that appropriate surface modification using PEG could effectively improve the biocompatibility of the prepared delivery carrier and moderate hemolytic toxicity in our study.

To establish causality between the potential acute toxicity observed with blood biochemistry testing and the associated pathological changes elicited by the nanoparticles in various organ systems, mice were sacrificed 8 h after intravenous administration of the formulations, and the heart, liver, spleen, kidneys, and lungs were subjected to standard hematoxylin and eosin (H&E) staining. As shown in Fig. 6L, no appreciable pathological findings were observed in the respective control saline/siSCR^Cy3^ and GO/siSCR^Cy3^ groups in agreement with our blood biochemistry results. However, non-PEGylated GO^PEI^ nanoparticles elicited evident and visible structural damage to the liver and kidney by disrupting the physiological histology of the organ parenchyma. This effect was partially mitigated by nanoparticle PEGylation in the GO^PEI^-mPEG group, although a certain degree of organ damage was still visible in the liver. We concluded that nanoparticle PEGylation considerably decreased the off-target toxicity caused by the cationic polymer component of the nanoparticle; however, due to the propensity of the majority of systematically administered nanoparticles to accumulate in the liver, the IHC results indicated that a certain degree of hepatotoxicity and nephrotoxicity was associated with GO^PEI^-mPEG administration.

Next, the in vivo anti-tumour and cisplatin chemosensitization effect of GO^PEI^-mPEG-siSOD1 was evaluated in subcutaneous A2780 and A2780^DDP^ cell-derived xenograft models (Fig. 6M). Relative to the A2780^DDP^ group, identical 0.5 mg/kg intratumoral cisplatin dose induced a significantly higher tumour growth inhibition rate (276.3 ± 72.39 mm^3^, *p* = 0.0170) in the sensitive A2780 tumours, confirming that the cisplatin-resistant phenotype of A2780^DDP^ was maintained in vivo as well. Further, we concluded that combined naked siSOD1 and cisplatin treatment had a negligible overall effect on tumour growth compared to cisplatin treatment only (770.81 ± 135.33 mm^3^, *p* = 0.9976). In addition, we found that intratumoral combined GO^PEI^-mPEG-siSCR and cisplatin treatment elicited significantly higher tumour growth inhibition relative to the 0.5 mg/kg naked siSOD1 plus cisplatin group (460.61 ± 59.32 mm^3^, *p* = 0.0046). This increased growth inhibition effect was attributed to the previously identified toxicity of the graphene nanoparticle on cancer cells observed with IHC and blood biochemistry testing, which despite the PEGylation, was not entirely circumvented. Finally, we found that relative to GO^PEI^-mPEG-siSCR group, 0.5 mg/kg siSOD1 complexed with GO^PEI^-mPEG at a 3:1 w/w ratio co-administered with cisplatin resulted in significant tumour growth inhibition (237.5799 ± 85.18 mm^3^, *p* = 0.0265) and structural tumour tissue damage (Fig. 6N) during the 14-day long therapeutic window. These results indicated that relative to the control siSCR group, GO^PEI^-mPEG-siSOD1 elicited partial re-sensitization of A2780^DDP^ xenografts to cisplatin, which was attributed to the *SOD1* knockdown via RNAi.

## Discussion

Post-treatment disease recurrence and cisplatin-resistance development are major therapeutic obstacles for ovarian cancer patients [2]. Hence, in this study we aimed at addressing this clinical phenomenon and prepared a GO^PEI^-mPEG siRNA nanocarrier to selectively target *SOD1* in vivo via RNAi, which we have previously identified as a potential target to reverse cisplatin-resistance [22]. GO^PEI^-mPEG exhibited favourable physicochemical characteristics for translational drug delivery applications regarding hydrodynamic diameter, ζ-potential, and PDI [24].

Following PEGylation, the cellular uptake studies confirmed the presence of adequate PEI surface charge to mediate endosomal escape via the intracellular proton sponge effect [42]. This mechanism was confirmed with fluorescent live cellular uptake studies, as an abrupt increase in fluorescence of siSCR^FAM^ was detected after 5 h indicating the detachment and regained fluorescence of siSCR^FAM^ following the endosomal escape. GO^PEI^-mPEG showed appropriate biocompatibility and formed homogenous solutions in various media modelling in vitro and in vivo conditions. At the same time, the gel- and fluorescence-based kinetic studies implied the complexed siSCR cargo was protected from RNase degradation. Further, all the prepared PEI-functionalized nanoparticles fully complexed siSCR at a 2:1 w/w ratio.

The cytotoxicity of the PEI cationic polymer component was significantly reduced with PEGylation; still, IHC imaging revealed that GO^PEI^-mPEG accumulation in the liver elicited structural damage and hepatotoxicity. We attributed the cytotoxicity produced by the PEI functional group to oxidative stress induction, which led to evident *SOD1* mRNA overexpression by GO^PEI^ in a concentration-dependent manner [43]. It can be concluded that non-PEGylated GO^PEI^ can counteract the knockdown effect mediated by siSOD1, which, theoretically based on our original hypothesis, may increase cisplatin resistance in surviving cell populations and subsequent passages via clonal selection.

The nanocarrier’s size, shape, surface charge, and surface functionalization determine the net toxicity of a graphene nanoparticle [44]. The increased ROS generation by concentration-dependent graphene treatment can cause oxidative stress-mediated mitochondrial damage and stress-reactive transcriptional factor activation, altering the gene expression profile of the affected cell [45]. The resultant oxidative stress, thus, may upregulate vital antioxidant enzymes, including but not limited to *CAT*, *PRX*, *SOD2*, and *SOD1*, as a defense mechanism [46]. For instance, Mendonca et al. showed that graphene oxide caused significant *SOD1* overexpression from 1 h up to 7 days following transfection [46]. A similar cellular effect was published by Jarosz et al., who found that both *SOD1* and *SOD2* were overexpressed upon treating HepG2 cells with GO in a concentration-dependent manner [47].

Further, we concluded that, like cisplatin, both GO^PEI^ and GO^PEI^-mPEG nanoparticles elicited mitochondrial dysfunction, evident from the specific activation of various arms of the UPR^mt^ pathways [48]. GO-based nanoparticles and PEI have been shown to impair tumour progression and suppress metastatic potential by inhibiting and disrupting mitochondrial respiration [49]. In addition, GO-induced ROS generation can stimulate mitochondrial dysfunction and subsequent apoptotic signalling pathways, which may explain the decrease in cisplatin IC_50_ values of A2780^DDP^ cells following GO^PEI^ treatment [47]. Through a series of immunohistochemistry analyses among various treatment groups to monitor the change in protein abundance of 7 UPR^mt^ signature genes including FOXO3, HSP60, LC3B, NRF1, SIRT3, SOD1, and SOD2 according to previous literature [48]. We have concluded that both GO^PEI^-mPEG mediated in vivo SOD1 knockdown evoke UPR^mt^ activation (detailed data not shown). Further, we observed the baseline upregulation of UPR^mt^ markers upon cisplatin treatment. To the best of our knowledge, this is the first report of cisplatin-induced activation of all three arms of the UPR^mt^ in ovarian cancer [49]. While further exploration of these pathways is beyond the scope of this study, based on the striking differences in the activation of UPR^mt^ pathways between baseline and upon cisplatin treated cells, it would be intriguing to explore further the potential involvement of these pathways in intrinsic or acquired cisplatin resistance in other cell lines as well. Furthermore, as graphene-based nanomaterials tend to accumulate and intercalate in the mitochondrial outer membrane causing mitotoxicity, thus it would be reasonable to examine whether the activation of the UPR^mt^ could be used as a reporter in graphene biosafety and biocompatibility assays [50].

## Conclusion

Despite the observed toxicities mediated by the respective nanoparticles, the in vivo results showed that *SOD1* knockdown has a partial chemo-sensitising effect in cisplatin resistance relative to the siSCR control group. This observed effect is per our previous in vitro results. We concluded that while these promising results exhibited the potential role of *SOD1* as a therapeutic target for the reversal of cisplatin resistance, yet the full therapeutic potential of *SOD1* knockdown has not been fully demonstrated in this study due to the *SOD1* mRNA induction properties of the nanoparticles, which counteracted the effect of the siSOD1 dosing primarily arising from the toxicity mediated presumably by the cationic polymer component. Overall, the therapeutic applicability of *SOD1* knockdown in reversing cisplatin resistance will be further investigated using other drug delivery platforms such as lipid nanoparticles (LNPs) and in other cell- and patient-derived xenograft models.

### Supplementary information

Figure Legend for Supplementary Figure 1

Supplementary Figure 1

## Data Availability

Materials described in this manuscript including all relevant raw data will be freely available from the corresponding author for non-commercial use upon request.
