# Supplementary material for: Targeting SOD1 via RNAi with PEGylated graphene oxide nanoparticles in platinum-resistant ovarian cancer
Source: Cancer Gene Ther. 2023 Aug 15;30(11):1554–68. doi: 10.1038/s41417-023-00659-2 (PMC10645591; doi:10.1038/s41417-023-00659-2)
Supplement: Supplementary file 1 — Figure Legend for Supplementary Figure 1 [file 41417_2023_659_MOESM1_ESM.docx]

**Supplementary Figure 1. (A, B)** The UV-vis spectra of GO, GO^PEI^, and GO^PEI^-mPEG were measured with and without siRNA complexation, and the absorbance peaks were observed at 260, 230, and 236 nm. UV-Visible spectroscopy was used to confirm the nanoparticle-siRNA electrostatic interactions. The absorbance spectra of GO^PEI^ (0.01 mg/ml), siSCR (2.5 µg/ml), and GO^PEI^-siSCR were measured. **(C)** Colloidal stability of GO^PEI^ was assessed in DIW, 0.9% NaCl, PBS, RPMI, RPMI + 10% FBS, 50% FBS, and RPMI + 10% FBS, 6 months after treatment. Nanoparticle aggregation was observed in GO^PEI^ + RPMI + 10% FBS. **(D)** Serum stability studies. A range of controls was used, including naked siSCR in DIW, siSCR in serum, different amounts of serum, nanoparticles in serum, and nanoparticles in water. The graph shows the fate of naked siSCR without a carrier in serum. **(E)** Laser Confocal Microscopy images of blank, siSCR^FAM^, GO^PEI^, Lipo^2000^, GO^PEI^-siSCR^FAM^ and Lipo^2000^-siSCR^FAM^. **(F)** Flow cytometry analysis. Fluorescence graphs (FL1-H gating FAM versus cell count) as observed in FACS, blank control, naked siSCR^FAM^, Lipo^2000^-siSCR^FAM^ (60 nM), Lipo^2000^- siSCR^FAM^ (90 nM), GO^PEI^- siSCR^FAM^ (60 nM) and GO^PEI^- siSCR^FAM^ (90 nM). **(G, H)** Determination of cisplatin IC_50_ values of A2780 and A2780^DDP^ cell lines with clonogenic assay.
