# Supplementary material for: Targeting SOD1 via RNAi with PEGylated graphene oxide nanoparticles in platinum-resistant ovarian cancer
Source: Cancer Gene Ther. 2023 Aug 15;30(11):1554–68. doi: 10.1038/s41417-023-00659-2 (PMC10645591; doi:10.1038/s41417-023-00659-2)
Supplement: Supplementary file 2 — Supplementary Figure 1 [file 41417_2023_659_MOESM2_ESM.pptx]

## Slide 1
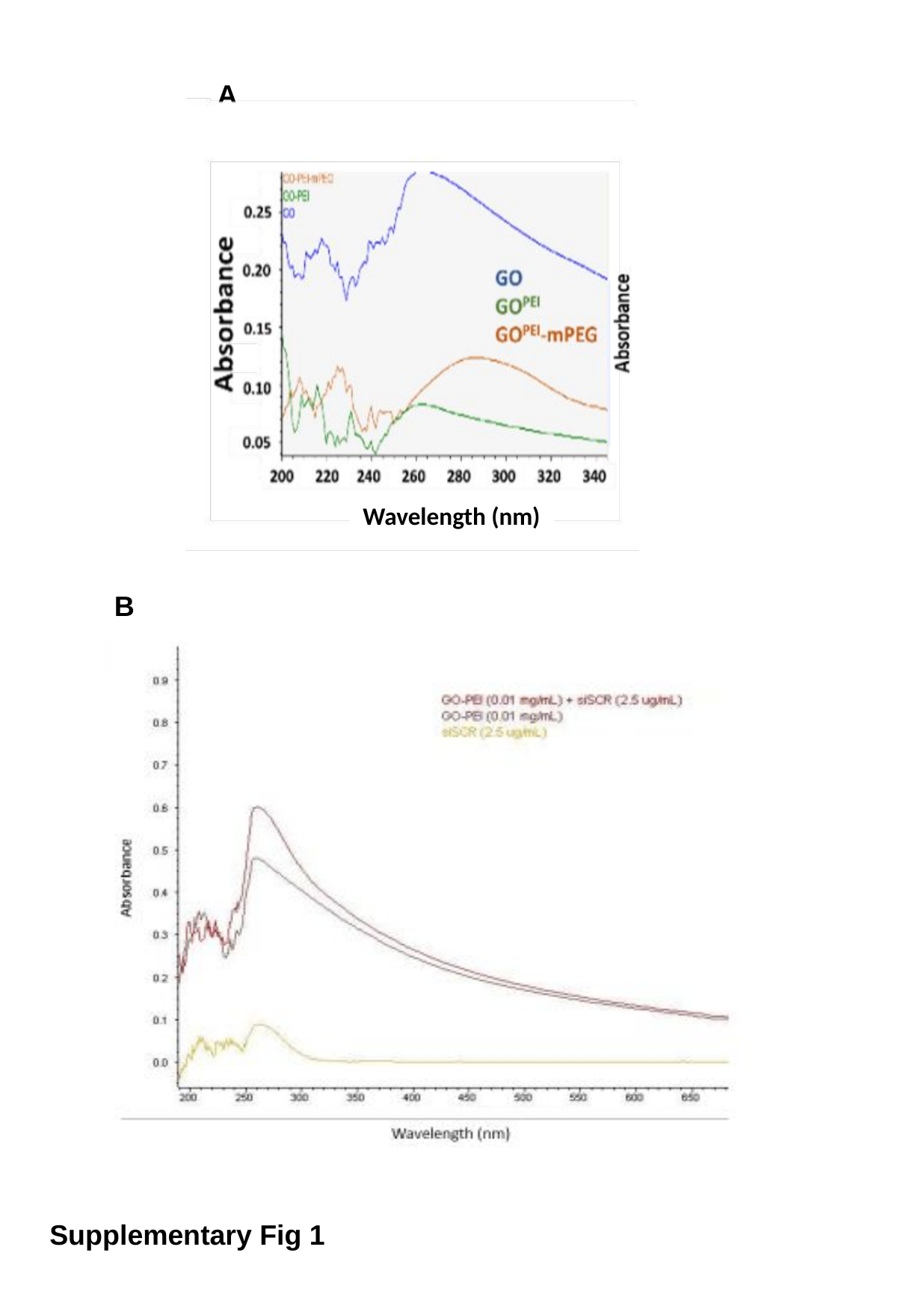

Wavelength (nm)
A
B
Supplementary Fig 1

## Slide 2
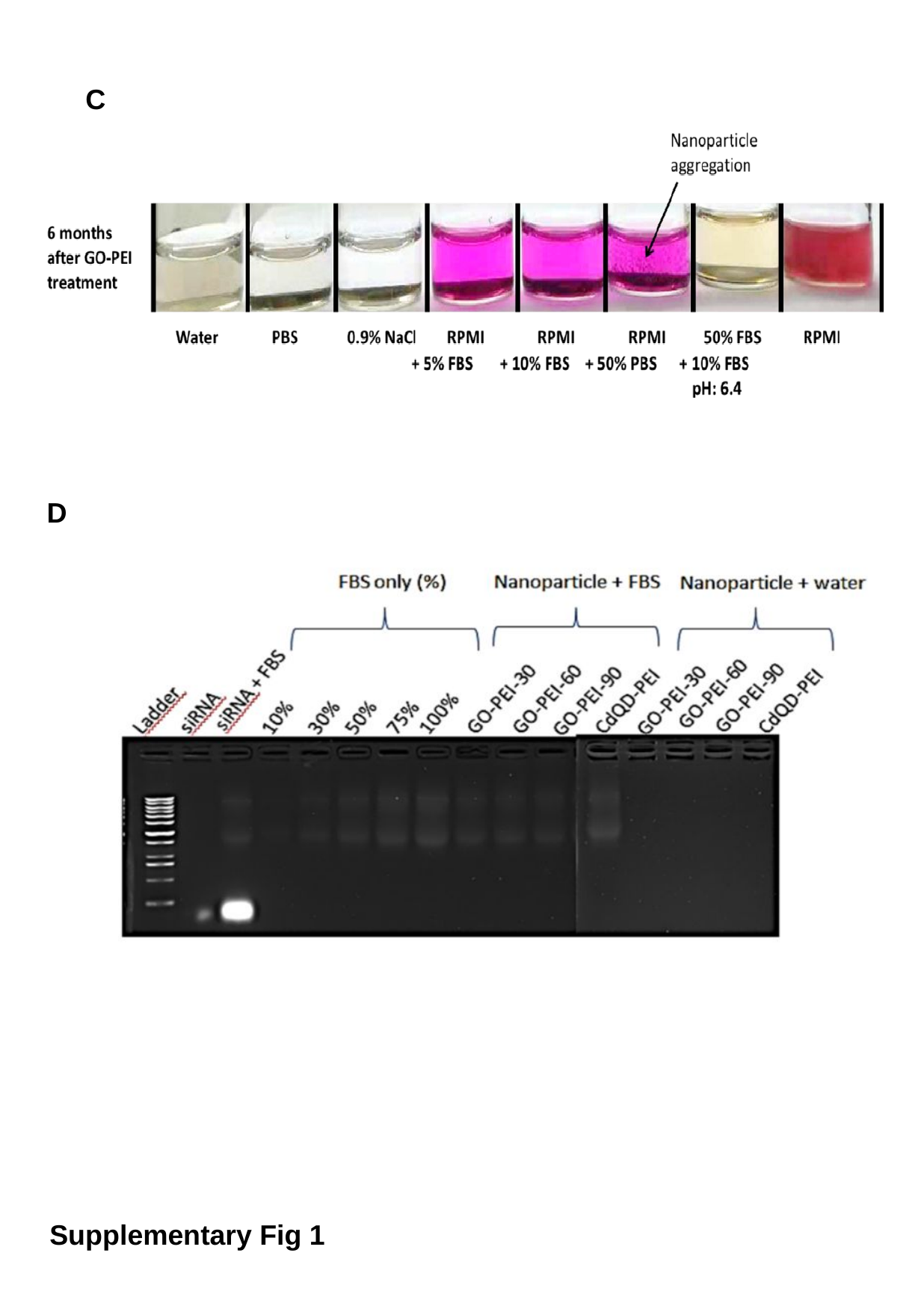

C
D
Supplementary Fig 1

## Slide 3
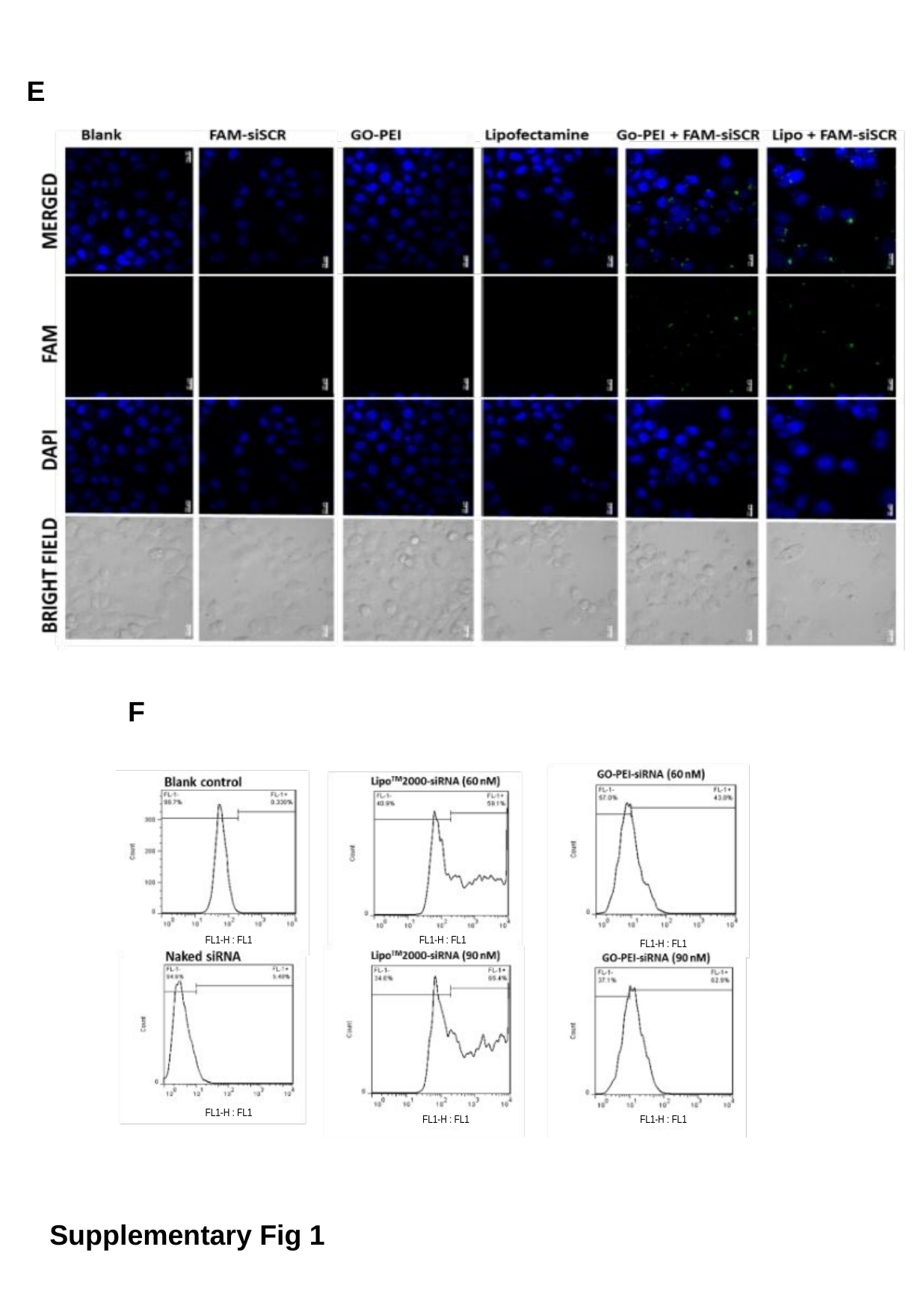

E
F
FL1-H : FL1
FL1-H : FL1
FL1-H : FL1
FL1-H : FL1
FL1-H : FL1
FL1-H : FL1
Supplementary Fig 1

## Slide 4
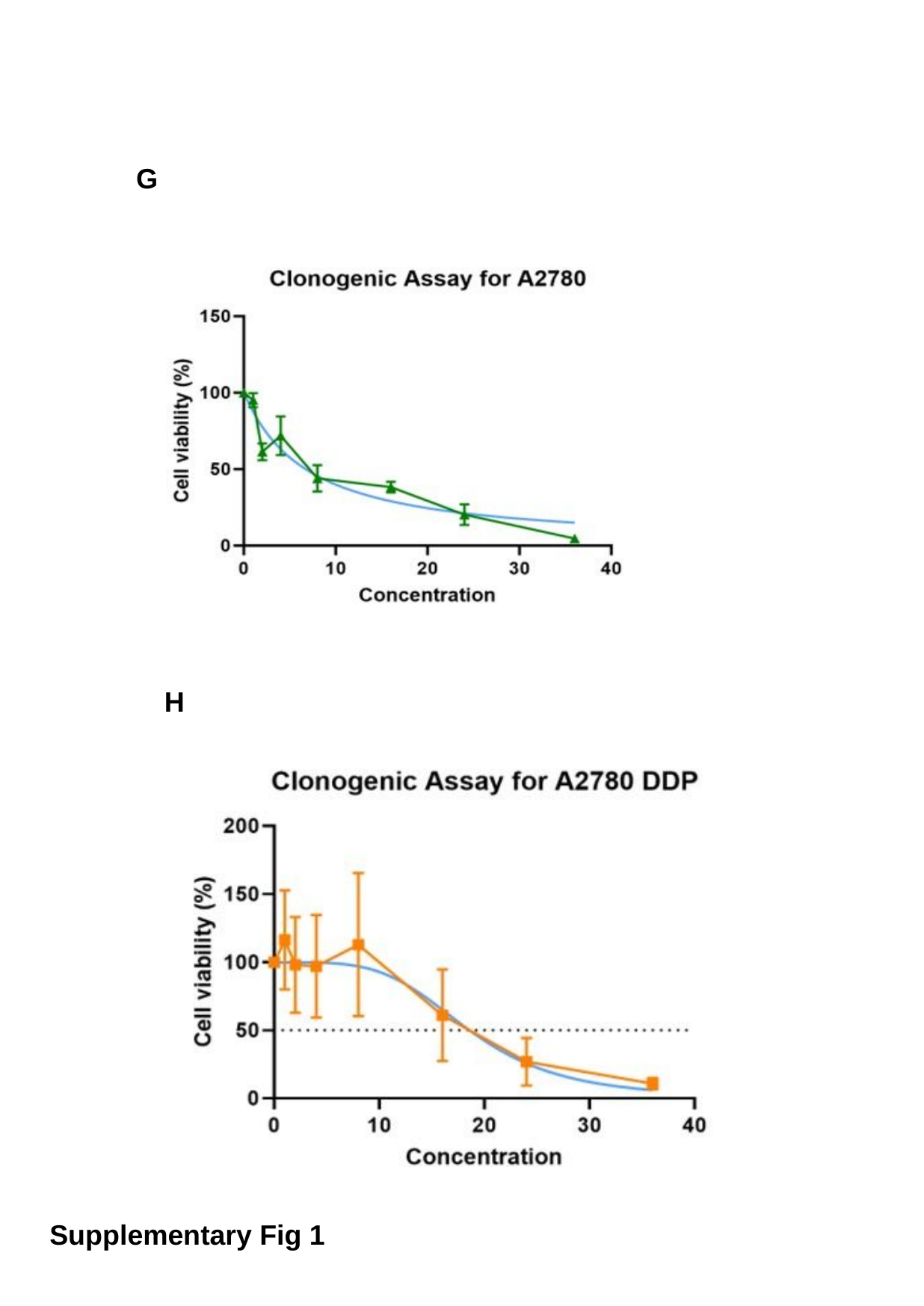

G
H
Supplementary Fig 1
